# Identification of major loci governing 13 agronomic traits and the fine-mapping of *CaSUN29* regulating fruit length in pepper

**DOI:** 10.1186/s43897-025-00179-7

**Published:** 2025-12-01

**Authors:** Yihao Wang, Lingkui Zhang, Xiaolong Yang, Feng Cheng, Bin Chen, Xiaofen Zhang, Sansheng Geng, Heshan Du

**Affiliations:** 1 State Key Laboratory of Vegetable Biobreeding, National Engineering Research Center for Vegetables, Key Laboratory of Biology and Genetics Improvement of Horticultural Crops (North China), Beijing Vegetable Research Center, Beijing Academy of Agriculture and Forestry Sciences, Beijing, 100097 China; 2 State Key Laboratory of Vegetable Biobreeding, Key Laboratory of Biology and Genetic Improvement of Horticultural Crops of the Ministry of Agriculture and Rural Affairs, Sino-Dutch Joint Laboratory of Horticultural Genomics, Institute of Vegetables and Flowers, Chinese Academy of Agricultural Sciences, Beijing, 100081 China

**Keywords:** Recombinant inbred line, Fruit length, IQD/SUN protein, Cell expansion, Trait-locus network

## Abstract

Pepper (*Capsicum* spp.), one of the world's most important vegetable and spice crops, has attracted considerable attention due to its phenotypic diversity, abundant secondary metabolites (particularly capsaicinoids), and substantial economic value. However, current research on the genetic basis and key regulatory genes underlying most traits in pepper is limited. Therefore, in this study, we used two parental lines that presented multiple phenotypic differences—namely, BVRC1 and BVRC25—to generate a recombinant inbred line (RIL) population (F_10_ generation, 216 lines), after which we performed whole-genome re-sequencing on all lines. Based on a high-resolution bin map, 19 significant loci were identified in association with 13 traits, explaining an average of 26% of the phenotypic variance in the RILs. On the basis of uncovering the major effect locus *FL-3.2* (*FS-3.1*) for fruit shape/fruit length, we constructed new genetic populations to finely map and clone a novel minor-effect regulatory locus *FL-10.1* for fruit length. Candidate gene *CaSUN29* encoded an IQD protein that was specifically expressed in the early stage of fruit development, affected cell expansion in the pepper pericarp, and subsequently impacted fruit elongation. Virus-induced gene silencing of *CaSUN29* in pepper resulted in shorter fruit, while heterologous *CaSUN29* overexpression in tomato led to longer fruit. In contrast to the pleiotropic locus *FL-3.2,* which regulated fruit length, width, and shape simultaneously, *FL-10.1* only regulated fruit length. Based on the identified loci, a trait–locus network was constructed to understand the correlations among traits based on the degree of locus linkage. These findings are helpful for our exploration of new genes regulating important traits, providing better understanding of the linkage relationships among complex traits.

**Supplementary Information:**

The online version contains supplementary material available at 10.1186/s43897-025-00179-7.

## Core

This study identified 19 key loci governing 13 agronomic traits in pepper through the high-resolution mapping of a recombinant inbred line population. A novel minor-effect locus, FL-10.1, was cloned, revealing that CaSUN29 (encoding an IQD protein) regulated fruit elongation by modulating cell expansion during early fruit development. Unlike the pleiotropic locus FL-3.2, FL-10.1 specifically controls fruit length, highlighting the distinct genetic mechanisms underlying fruit morphology. The constructed trait–locus network elucidates the genetic linkages among complex traits, offering molecular insights for precision breeding and enhancing yield-related traits in pepper.

## Gene & accession numbers

The gene information utilized in this study is accessible on the SolPGD (http://www.bioinformaticslab.cn/files/genomes/pepper_pan/genome_v1/Canb/) under the following accession numbers: CaSUN29 (Canq10g001705).

## Introduction

Peppers are cultivated extensively worldwide and are currently the vegetable crop with the largest cultivation area in China (FAOSTAT 2023). *Capsicum annuum* L. is the primary cultivated species, and it is characterized by rich genetic resources and significant phenotypic variation, with more than 12 distinct types classified solely based on fruit shape (Paran and van der Knaap 2007; Borovsky et al. 2022; Cao et al. 2022). Peppers destined for different regions and applications have different trait-based requirements, such as factors related to their shape and pungency. The objective of modern pepper breeding has gradually evolved in individualized directions (Ahmar et al. 2020; Tiwari et al. 2022). Therefore, revealing the genetic basis of important agronomic traits facilitates molecular-assisted breeding for the selection of pepper varieties that meet different market demands.

Most yield- or fruit-related traits in pepper are quantitative traits, which are typically regulated by multiple loci or genes. The majority of these are still at the quantitative trait locus (QTL) identification stage,with only a few having been cloned. *fs3.1* and *fs10*, which encode a TONNEAU 1 Recruiting Motif (TRM) protein and an Ovate Family Protein (OFP), respectively, are crucial loci regulating fruit shape. *fs3.1* regulates the elongation and narrowing of pepper fruit, while *fs10* adjusts the fruit shape index (the ratio of fruit length to fruit width) to a value closer to 1, indicating that they have opposing regulatory effects on fruit shape (Colonna et al. 2019; Borovsky et al. 2022; Cao et al. 2022). *fs3.1* and *fs10* are also the only two loci regulating fruit length that have been cloned, although they also simultaneously regulate fruit width. To date, no loci that solely regulate pepper fruit length have been cloned. This is similar to the investigation in tomato, in which loci, such as *SUN*, *fs8.1*, and *OVATE*, regulate fruit shape, primarily through variations in the number of cells along distinct growth axes, either promoting or suppressing fruit elongation (Liu et al. 2002; Xiao et al. 2008; Wang et al., 2019; Zhu et al. 2023). There are also some loci in tomato, such as *fas* and *lc*, that regulate fruit shape by controlling the number of fruit locules(Cong et al. 2008; Muños et al. 2011). In pepper, many loci for the number of fruit locules, such as *lcn1.1*, *Nlo2.1*, *Nlo8.1*, *Nlo12.1*, and *Nlo12.2*, have been identified (Barchi et al. 2009; Ma et al., 2022). Among these loci*,* only *lcn1.1*, which encodes the BREVIS RADIX (BRX) protein, has been cloned (Ma et al., 2022). Several loci have been identified as regulating fruit weight in pepper, including *FW1*, *FWE2.1, FW2.2*, *fw4.1, FW6.1*, *FW6.2*, *qFWT7.1* and *qFWT7.2 *(Chaim et al. 2001; Zygier et al. 2005; Han et al. 2016; Liu et al., 2022; Lopez-Moreno et al., 2023). However, the key regulatory genes have not yet been cloned. Leaf size plays a critical role in photosynthesis and respiration, ultimately influencing the final pepper yield (Lawson and Milliken 2023). Several QTLs related to leaf size, including *LL-11.1*, *LL-11.2*, *LW-8*, *LfA1.1*, *LfA2.1*, and *LfA3.1*, have been identified (Yarnes et al. 2013; Han et al. 2016; Chunthawodtiporn et al. 2018). The regulatory genes of some quality traits in peppers, such as *CCS* for mature fruit color and *Pun1, Pun2* and *Pun3* for the presence or absence of capsaicinoids, have been cloned (Popovsky and Paran 2000; Stewart et al. 2005; Han et al. 2019; Yi et al. 2022). Due to the current lack of effective means for verifying the functions of these loci or genes in peppers, the accuracy and genetic linkages of these loci in molecular breeding require verification from multiple aspects, such as consensus loci identified using diverse populations and approaches (Zhang et al. 2020; Wu et al., 2022). Relevant studies on trait genetic networks in crops, such as tomato and soybean, play a significant role in molecular-assisted breeding and the aggregation of target traits (Fang et al. 2017; Zhang et al. 2018), indicating strong or weak linkage relationships among traits.

The IQ67 DOMAIN (IQD)/SUN family is a class of calcium-regulated binding proteins that primarily integrates CaM-dependent calcium signaling, and potentially other signaling pathways, to modulate cell shape and growth in *Arabidopsis thaliana *(Bürstenbinder et al. 2017a, 2017b), and it has been demonstrated to be associated with fruit morphogenesis in various crops (Xiao et al. 2008; Pan et al. 2016; Dou et al., 2018; Cheng et al. 2021). In tomato, *SUN*(*IQD12*) was the first identified IQD family gene related to fruit shape, functioning in tomato fruit elongation by reducing the number of cells in the mediolateral septum and increasing the number of cells in the proximal–distal direction of the fruit pericarp (Xiao et al. 2008; Wu et al. 2011). Subsequently, the *SUN* gene was identified as a key gene regulating fruit shape in cucumber (Pan et al. 2016). The homologous gene of *IQD26* has also been identified as an important gene regulating fruit shape in both watermelon and wax gourd (Dou et al., 2018; Cheng et al. 2021), and its interaction with map65-1 regulates the direction of cell division in tomato fruit, thereby affecting fruit morphogenesis (Bao et al. 2024). At present, 33 IQD/SUN family genes have been identified in tomato (Huang et al. 2013). Among these genes, three have been found to be related to fruit morphology, possessing pleiotropic effects and influencing both fruit length and width (Xiao et al. 2008; Bao et al. 2023, 2024). No IQD/SUN family genes have been cloned in pepper.

Compared to traditional F_2_ populations, recombinant inbred line (RIL) populations are an effective tool for multi-trait mapping, because they are genetically stable and can provide many materials with rich variations. However, the amount of time required for the establishment of a stable RIL population is considerable, resulting in there being a limited number of related studies in pepper (Han et al. 2016; Liu et al., 2022; Lee et al., 2024). In contrast to the traditional molecular marker detection of QTL regions, genome-wide association studies (GWAS) utilize a wide range of single nucleotide polymorphisms (SNPs) across the whole genome, enhancing the mapping resolution of complex traits. Generally, the strong population structure of natural populations affects the statistical power of GWAS (Huang et al. 2011; Wei et al., 2024). Hybrid populations, such as RILs, have also been developed for GWAS, thereby eliminating the effects of population structure in these analyses (Buckler et al. 2009; Korte and Farlow 2013).

In this study, we established a high-generation RIL population and re-sequenced 216 lines. A high-density bin map composed of 21,306 bin markers was then constructed. Through the analysis of the phenotypic variations of 13 agronomic traits, 19 significant loci were identified. We also discovered a novel locus, *FL-10.1*, that solely regulates fruit length without influencing fruit width, and we found that candidate gene *FL-10.1* encodes an IQD family protein. Based on these results, a trait–locus network was constructed to reveal the strength of linkages among loci for different traits. The current findings help understand the complex linkage among traits and support the aggregation of desirable traits in pepper breeding, providing new insights for research on pepper fruit length.

## Results

### Construction of a high-density bin map using an RIL population

**Fig. 1:**
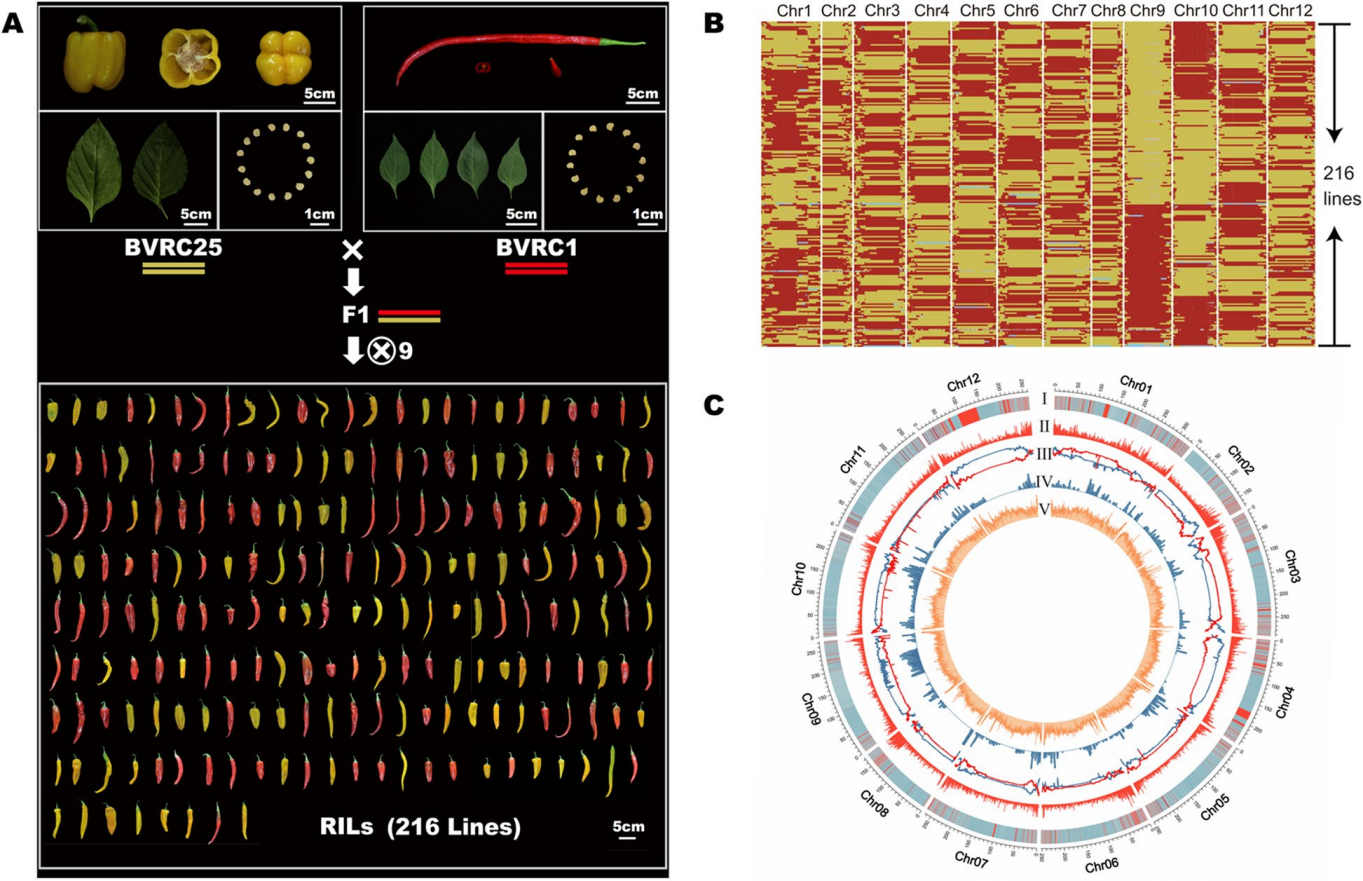
Construction and re-sequencing analysis of the recombinant inbred line (RIL) population. **A** RIL population generated by two high-generation pepper inbred lines BVRC1 and BVRC25. **B** Recombination bin map of 216 RILs after clustering by genotype. Different colors indicate different genotypes: red, BVRC1; yellow, BVRC25; blue, heterozygous; gray, not available. **C** Genome-wide distribution of single nucleotide polymorphisms (SNPs), GC content, and number of genes between two parental lines and the recombination density in the RIL population. I, bin recombination frequency; II, SNP density; III, bin’s parental distribution; IV, gene density; V, GC content

To investigate the molecular basis underlying trait differences among pepper varieties, an RIL population was developed using two self-bred parental lines, namely, BVRC1 (genotype: AA) and BVRC25 (genotype: BB), as representative genetic resources (Fig. 1A). These lines exhibit striking phenotypic differences: BVRC1 produces mature fruit that are red, slender, and linear, with an average weight of 15–20 g, whereas BVRC25 yields yellow, blocky fruit weighing 160–200 g each. An F_2_ segregating population comprising 220 plants was generated by crossing BVRC1 and BVRC25, from which 216 lines were selected and subjected to nine generations of selfing to develop a stable RIL population, which presented a variation amplitude of fruit length ranging from 7.0 to 28.8 cm,while that of the fruit shape index varied from 1.36 to 11.41. Such extensive phenotypic variability provides a robust foundation for subsequent genetic analysis of phenotypic diversity. To comprehensively evaluate this variability, a 2-year field trial was performed in Beijing in 2021 and 2023, and 13 traits were systematically measured in the two parental lines (Table S1). These traits included eight fruit-related characteristics (fruit shape, fruit length, fruit width, fruit tip, fruit locule number, mature fruit color, fruit weight, and presence of capsaicinoids), three leaf-related traits (leaf area, leaf length, and leaf width), and two seed-related traits (seed area and seed weight). All 13 traits showed significant differences between the parental lines (Table S2). Moreover, most of the traits measured in the RIL population exhibited high correlations across biological replicates and growing seasons, underscoring their stability under diverse environmental conditions (Table S3).

The whole genomes of the two parental lines were sequenced at a 30-fold depth, and each of the 216 RILs was sequenced at an average fivefold depth, generating 4,382 GB of clean data. (Table S4). A comprehensive analysis identified 13,997,685 SNPs corresponding to an average SNP density of 4666 SNPs/Mb between the parental lines. Each RIL contained an average of 12,746,600 SNPs. A sliding-window approach with a window size of 15 SNPs was subsequently employed, sliding 1 SNP at a time to determine the genotype for each window. This method enabled us to infer genotypes for each RIL, resulting in the identification of 21,306 bin markers across all 216 RIL lines (Figure S1 and Table S5). The recombination frequencies in the RILs ranged from 0.217 to 0.749 (Table S6). Based on the binmap and distribution of SNP sources, 99% of the regions on the chromosomes were homozygous, suggesting that the RIL population was genetically stable (Fig. 1B and Table S5). The genome-wide SNP distribution analysis revealed that although SNPs between the two parents were relatively evenly distributed throughout the genome, the distribution of recombination frequency showed a strong bias towards the ends of all 12 chromosomes, which were highly correlated with the gene distribution, except for a region on chromosome 9 (Fig. 1C). Principal component analysis (PCA) was performed on the RIL population, and the RILs were clustered based on their genotype. Both analyses demonstrated that the RIL population had a less distinct population structure at the genomic level (Figure S2).

### Efficient identification of major loci and regulatory genes using GWASRIL method

**Fig. 2:**
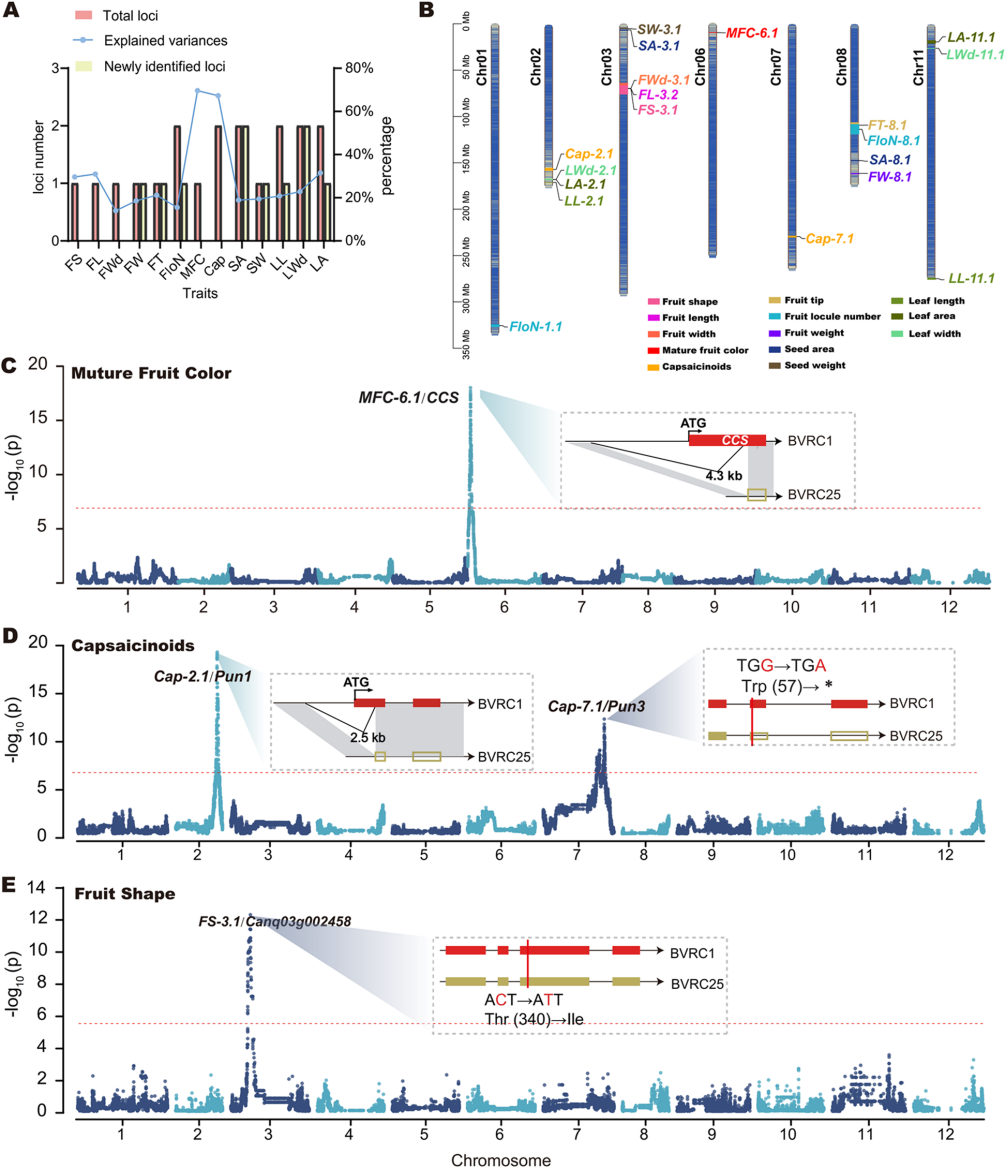
Identification of 19 loci associated with 13 agronomic traits. **A** Summary of locus identification for 13 agronomic traits. The explained variances take the maximum value among the loci for each trait. FS: fruit shape; FL: fruit length; FWd: fruit width; FW: fruit weight; FT: fruit tip; FloN: fruit locule number; MFC: mature fruit color; Cap: capsaicinoids; SA: seed area; SW: seed weight; LL: leaf length; LWd: leaf width; LA: leaf area. **B** Physical positions of all identified loci on the Qiemen pepper genome. **C** Manhattan plots for mature fruit color and sequence variation of *CCS* between BVRC1 and BVRC25. **D** Manhattan plots for capsaicinoid presence or absence and sequence variations of *Cap-2.1* (*Pun1*) and *Cap-7.1* (*Pun3*) between BVRC1 and BVRC25. **E** Manhattan plots for fruit shape and sequence variation of *FS-3.1* (*Canq03g002458*) between BVRC1 and BVRC25. The hollow squares on the BVRC25 sequence indicate the loss of function of the corresponding genes

The whole-genome sequencing data were further used for GWAS (GWAS_RIL_). A total of 19 significant association loci were identified for 13 traits, of which seven overlapped with reported genes, two overlapped with reported QTLs, and 10 loci were not characterized (Figs. 2A and S3 and Table S7). These loci explained an average of 26% of the phenotypic variation for each trait, ranging from 14% for fruit width to 70% for mature fruit color (Fig. 2A). The physical positions of these loci on the pepper genome are detailed in Fig. 2B and Table S7. High-resolution mapping enabled us to identify key loci with known gene associations and uncover novel candidates for further investigation. Below, we detail the loci localization results for the four major fruit traits analyzed.

Among the identified loci, locus *MFC-6.1 (P* = 9.55e − 19) was significantly associated with mature fruit color in pepper. This locus covered a bin region of about 300 kb on chromosome 6, containing 25 genes, including a gene (*Capsanthin/Capsorubin synthase*, *CCS*) encoding a key enzyme involved in capsanthin/capsorubin synthesis, which was responsible for the red color of mature pepper fruit (Lefebvre et al. 1998) (Fig. 2C). A 4.3-kb deletion was identified upstream of the transcription start site and within the gene itself for *CCS* in BVRC25, resulting in the functional loss of *CCS* (Figs. 2C and S4). Further sequence analysis of each line showed that this deletion was specifically present in the mature yellow fruit lines of the RIL population and co-segregated with the mature yellow fruit phenotype, which was consistent with a previous report (Popovsky and Paran 2000).

An association analysis, based on capsaicinoid presence or absence identified significant loci on chromosomes 2 (*Cap-2.1*, *P* = 8.23e − 21) and 7 (*Cap-7.1*, *P* = 2.12e − 07) (Fig. 2D). Further analysis revealed that *Cap-2.1* encompassed the reported gene *Pun1*, which was a structural gene in the final step of capsaicinoid biosynthesis. Using known primers, we confirmed that the non-pungent parental line BVRC25 carried the *pun1-1* allele, which was characterized by a large loss-of-function deletion and was consistent with previous reports (Figure S5B) (Han et al. 2013; Gulzar et al. 2023). The *Pun3* gene, which encodes a MYB31 transcription factor that regulates capsaicin biosynthesis, was found at the *Cap-7.1* locus. Sequencing revealed that in BVRC25, the coding region of *Pun3* had an SNP leading to a nonsense mutation (Figures S5C and S5D), which was consistent with a previous report (Han et al. 2019). Our results thus suggest that the presence or absence of capsaicinoids in the RIL population is jointly regulated by variations in both the *Pun1* and *Pun3* genes.

The fruit locule number is a complex trait regulated by multiple loci. Two significant loci regulated the fruit locule number and explained about 30% of the phenotypic variation in this RIL population. Both loci overlapped with reported QTLs. *FloN-1.1* (*P* = 6.62e − 07) contains *CaBRX*, a gene that regulates the fruit locule number in pepper and has been cloned (Ma et al., 2022). However, *FloN-8.1* (*P* = 4.92e − 08) has been reported several times, but it has not yet been cloned. In this study, the *FloN-8.1* locus covered a 10.9-Mb region and contained 75 genes. Comparative analysis of the different genotypes at these loci in the RILs showed that both had a significant impact on the fruit locule number (Figure S6).

In terms of fruit shape, a significant locus, *FS-3.1* (*P* = 3.43e − 13), on chromosome 3 was consistently associated with fruit length, fruit width, and overall fruit shape in this RIL population. This locus covered a bin region of about 4.25 Mb on chromosome 3 and contained 25 genes (Table S7). Four of the genes exhibited non-synonymous mutations. Among them, our analysis identified an SNP that caused a non-synonymous mutation in *Canq03g002458* and that was significantly associated with the variation in fruit shape, fruit length, and fruit width in the RIL population. It has been reported as a strong candidate gene for *FS-3.1 *(Cao et al. 2022) (Figs. 2D and S7).

In addition, the following loci were also identified: *FT-8.1* (*P* = 4.03e − 07) for fruit tip, *FW-8.1* (*P* = 1.05e − 05) for fruit weight, *SA-3.1* (*P* = 5.68e − 07) and *SA-8.1* (*P* = 5.39e − 07) for seed area, *SW-8.1* (*P* = 2.32e − 08) for seed weight, *LL-2.1* (*P* = 1.06e − 06) and *LL-11.1* (*P* = 2.78e − 07) for leaf length, *LWd-2.1* (*P* = 1.92e − 07) and *LWd-11.1* (*P* = 9.49e − 06) for leaf width, and *LA-2.1* (*P* = 3.37e − 07) and *LA-11.1* (*P* = 2.55e − 07) for leaf area. Except for the identification of a known locus in leaf length (*LL-11.1*) and leaf area (*LA-2.1*), the remaining loci were all identified for the first time in this study. The major loci for seed area and seed weight were both located on chromosome 8, with explained variances of 18.9 and 19.5%, respectively. Based on the analysis of phenotypic variation of two seed area loci in the RIL population, *SA-3.1* was found to be a positive regulatory locus for the BB phenotype, while *SA-8.1* was a negative one (Figure S9). The locus for leaf area and leaf width on chromosome 2 could be pleiotropic, co-regulating these two traits, similar to fruit shape locus *FS-3.1* (Fig. 2B and Table S7).

### Novel locus FL-10.1 exclusively influences pepper fruit length

**Fig. 3:**
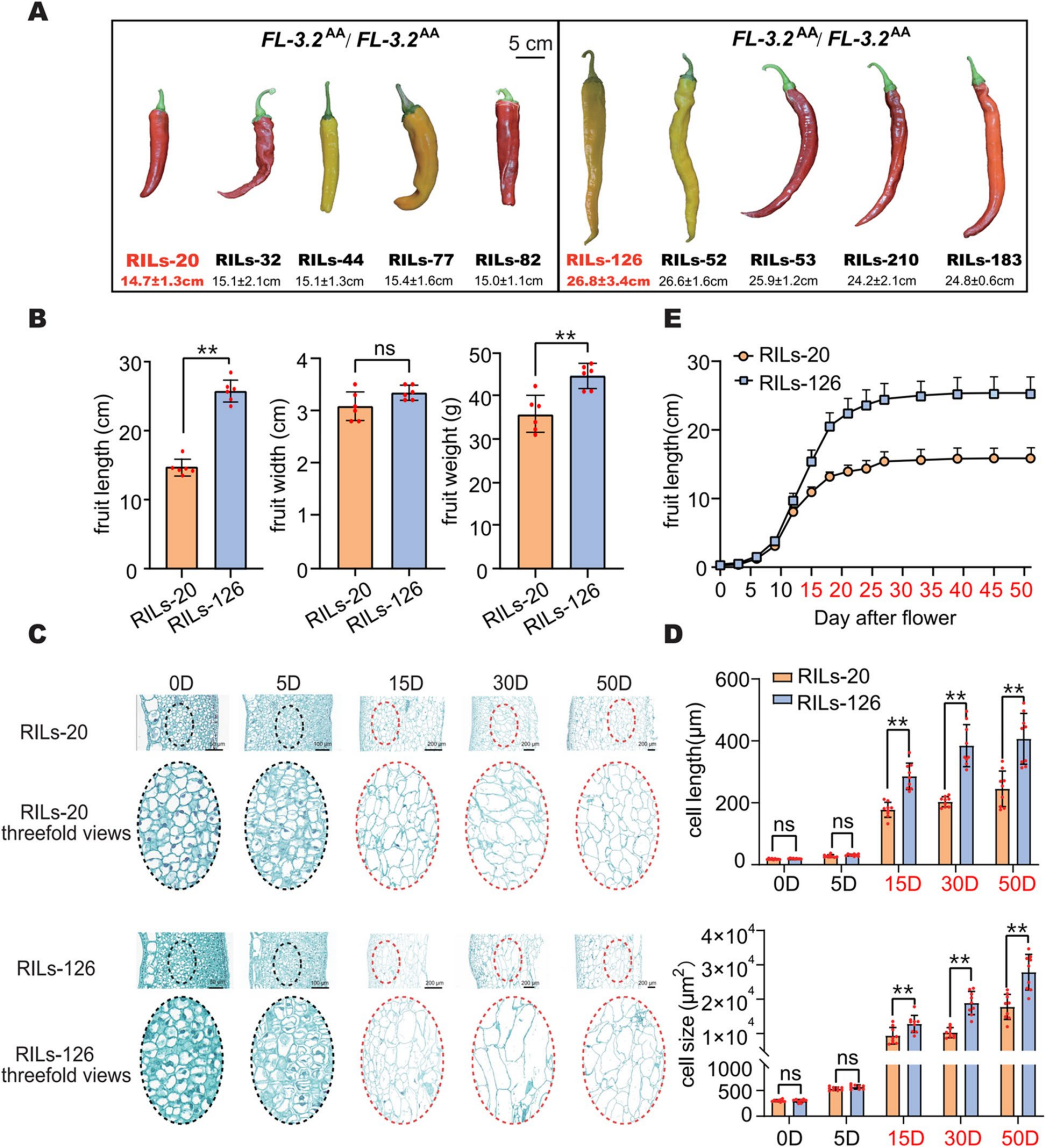
Fruit phenotypic differences and cytological observation between RILs-20 and RILs-126, which both carry the *FL-3.2*.^*AA*^ locus. **A** Both RILs-20 and RILs-126 carry the *FL-3.2* locus but exhibit significant differences in fruit length. **B** Differences in fruit length, width, and weight between RILs-20 and RILs-126. **C** Longitudinal sections of RILs-20 and RILs-126 at different stages of fruit (ovary) development. **D** Cell size and length of RILs-20 and RILs-126 at different fruit (ovary) development stages. **E** Fruit length of RILs-20 and RILs-126 at different fruit (ovary) development stages. The fruit development periods marked in red indicate the existence of significant differences. Values are presented as the mean ± SD. Student’s *t-*test was used to identify significant differences between the two groups (*, *P* < 0.05; **, *P* < 0.01; ns, no significant differences)

As mentioned previously, the locus *FL-3.2*, which regulates fruit length, was identified in this RIL population and explained approximately 30% of the phenotypic variation (Fig. 2A). However, we subsequently observed significant differences in fruit length among several lines carrying the *FL-3.2*^*AA*^ locus, indicating the potential existence of additional loci regulating fruit length (Fig. 3A). Therefore, two lines, i.e., RILs-20 (14.7 ± 1.3 cm) and RILs-126 (26.8 ± 3.4 cm), carrying the *FL-3.2*^*AA*^ locus but showing significant differences in fruit length, were selected for the analysis of fruit length variation and the identification of novel loci for fruit length (Fig. 3A). After clustering the RIL lines based on their genotypes, it was found that RILs-20 and RILs-126 had a closer genetic relationship and background (Figure S9). RILs-20 and RILs-126 showed no significant differences in fruit width, but the fruit weight of RILs-126 was significantly higher than that of RILs-20 (Fig. 3B). Considering the fruit length at different developmental stages and examining the pericarp at key intervals (0, 5, 15, 30, and 50 d), the most substantial difference in fruit length occurred between 5 and 15 d after development, primarily due to differences in cell length (Figs. 3C–E).

**Fig. 4:**
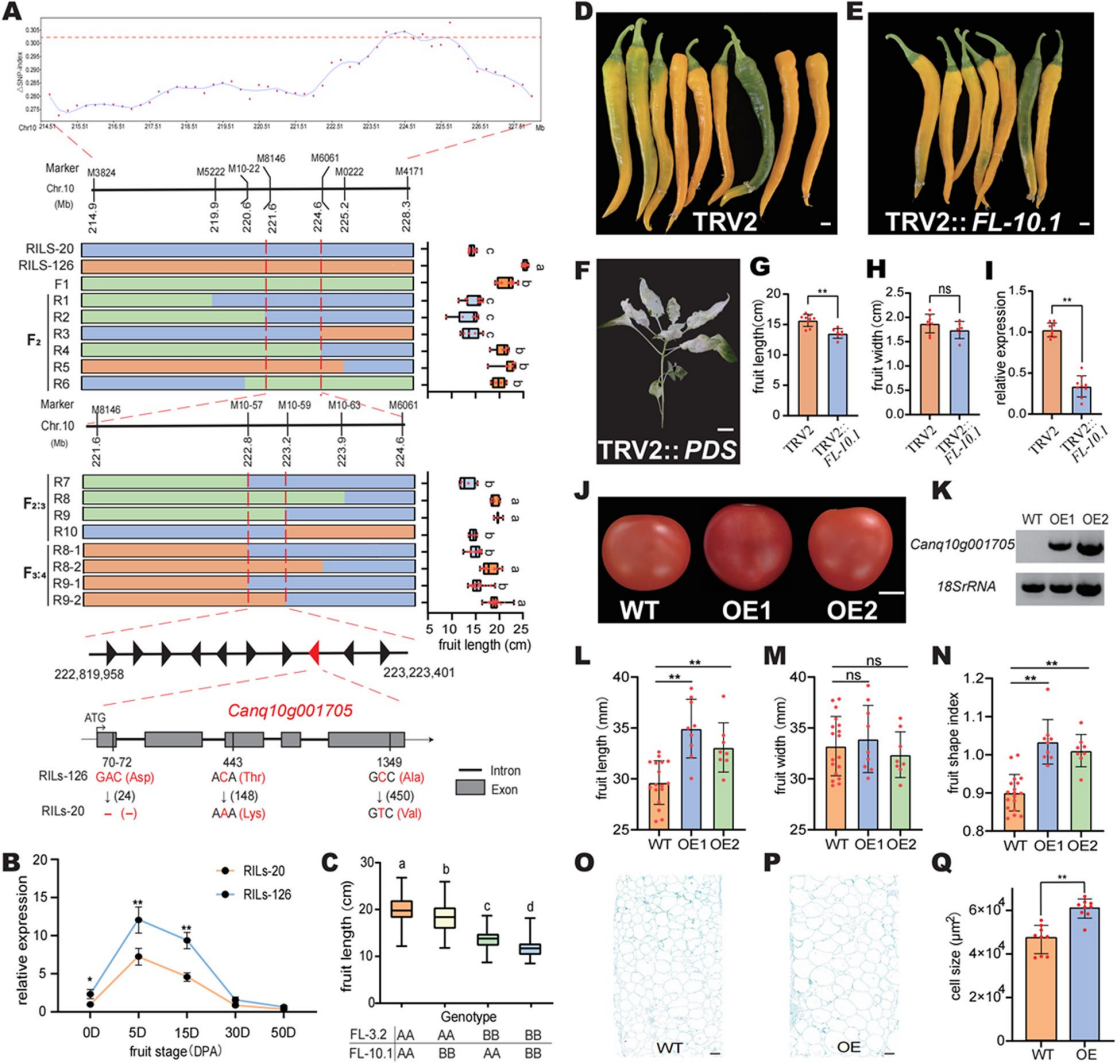
Fine mapping and functional analysis of *FL-10.1*. **A** Fine mapping results for *FL-10.1* and the sequence variations in *Canq10g001705* between RILs-20 and RILs-126. In the graphical genotype for critical recombinants, the blue square represents the genotype consistent with RILs-20; the orange square represents the genotype consistent with the RILs-126; and the green represents the genotype consistent with F_1_. R1-9 are representative recombinants. **B** Relative expression levels of *Canq10g001705* at different fruit (ovary) development stages between RILs-20 and RILs-126. **C** The effects of different genotype combinations of *FL-10.1* and *FL-3.2* on fruit length in the RIL population. **D** Fruit of pepper plants infected with the empty TRV2 vector. **E** Fruit of pepper plants infected with the TRV2:: *Canq10g001705* vector. **F** Pepper plants infected with the TRV2:: *PDS* vector. **G** Fruit length of TRV2-treated and *Canq10g001705-*silenced plants. **H** Fruit width of TRV2-treated and *Canq10g001705-*silenced plants. **I** *Canq10g001705* expression analysis in the fruit of TRV2-treated and *Canq10g001705-*silenced plants. **J** Phenotype of transgenic tomato fruit. WT, wild type; OE1 and OE2, *Canq10g001705*-overexpressing tomato lines. **K** Semi-quantitative analysis of *Canq10g001705* expression. 18S rRNA, internal reference. **L**-**N** Fruit length, fruit width, and fruit shape index, respectively, of the WT and transgenic lines. **O**-**P** Longitudinal sections in the mesocarp of WT and OE fruit at the mature stage. **Q** Cell size in the mesocarp of WT and OE fruits at the mature stage. Scale bars, 1 cm in D, E, F, and J, and 200 μm in O, and P. Values are presented as the mean ± SD. Student’s *t*-test was used to identify significant differences between the two groups (*, *P* < 0.05; **, *P* < 0.01). Tukey’s honestly significant difference (HSD) test is used to identify significant differences among multiple groups, and different letters above the boxes indicate statistically significant differences, *P* < 0.05

To map additional loci associated with fruit length, an F_2_ population (*n* = 617) was developed, and bulked segregant analysis (BSA) sequencing was performed on plants exhibiting extreme fruit lengths. This approach identified a significant candidate region on chromosome 10 (Fig. 4A). Initially, locus *FL-10.1* was located in the region flanked by 2 insertion–deletion (InDel) markers, 3824 and 4171. Through continuous screening of critical recombinants from F_2_ and F_2:3_ populations within this region, *FL-10.1* was precisely mapped to the interval between 222.82 and 223.22 Mb, where 10 genes were annotated (Fig. 4A). The fruit length of recombinants in the F_3:4_ populations also supported that *FL-10.1* was located within this interval (Fig. 4A). Based on sequence variation analysis among these 10 genes, *Canq10g001702* and *Canq10g001704* had non-synonymous variation, and *Canq10g001703* had three non-synonymous variations in coding sequences between RILs-126 and RILs-20 (Figure S10). The InDel and SNPs between the two parents were found within the coding region of *Canq10g001705*, resulting in a non-frameshift mutation and missense mutations (Figs. 4A and S11).

We collected the pericarps of RILs-126 at the developmental stages of 0, 5, 15, 30, and 50 d for transcriptome analysis and combined the gene expression data with the fruit length growth rate for K-means clustering (Figure S12A). The clustering results showed that the genes exhibiting expression trends analogous to the fruit length growth rate of RILs-126 were predominantly grouped in Cluster 2, encompassing 2008 genes (Figure S12B). Expression analysis of the genes within the *FL-10.1* candidate region revealed that four genes (*Canq10g001698*, *Canq10g001699*, *Canq10g001703*, and *Canq10g001704*) were not expressed during fruit development, while two of the remaining six genes (*Canq10g001701* and *Canq10g001705*) showed expression patterns consistent with Cluster 2 (Figure S13). *Canq10g001705* was highly expressed in pepper fruit, especially in the fruit pericarp during early fruit development (Figure S14). In RILs-20 and RILs-126, there was a significant difference in expression in the fruit pericarp from 5–15 d of fruit development, and the main difference in fruit length also occurred during this stage (Figs. 3E and 4B). Therefore, combining expression analysis and sequence variation analysis, the *Canq10g001705* gene was considered a strong candidate gene for *FL-10.1,* which encoded a calcium-dependent protein CaSUN29 belonging to the IQD family.

Enrichment analysis of Cluster 2 revealed that a substantial number of genes were enriched in Gene Ontology (GO) terms, such as “cell cycle process” and “regulation of cell cycle,” and Kyoto Encyclopedia of Genes and Genomes (KEGG) pathways, including “cutin, suberine, and wax biosynthesis” and various amino acid metabolic processes (Figures S12C and S12D). This suggests that the genes within Cluster 2 may be closely associated with fruit growth and development. Candidate gene *FL-3.2* was also present in Cluster 2. Transcription factors within Cluster 2 were also identified, and genes deemed to be minimally expressed in fruit (average FPKM < 2) were filtered out. A total of 51 transcription factors that exhibited co-expression with the fruit growth rate were selected, among which the bHLH and MYB families were the most prevalent (Figures S12E and S12F).

To determine the function of *Canq10g001705* in fruit morphology, *Canq10g001705* was silenced in pepper using the virus-induced gene silencing (VIGS) method with vacuum infiltration of seedlings. Plants infected with the TRV2 and TRV2::*PDS* vectors were treated as the negative and positive controls, respectively (Figs. 4D and F). Phenotypic investigation showed that the fruit length of the plants infected with the TRV2::*FL10.1* vector was significantly shorter than that of the plants infected with the empty TRV2 vector, while there were no pronounced differences in fruit width between them (Figs. 4D, E, G and H). qRT-PCR revealed that the *FL-10.1* expression level was significantly suppressed in the fruit of silenced plants infected with the TRV2::*FL10.1* vector compared to those infected with the empty vector (Fig. 4I). *Canq10g001705* was also heterologously overexpressed in tomato by *Agrobacterium tumefaciens*-mediated transformation. Two independent positive transgenic lines (OE1 and OE2) were generated, and these transgenic lines were verified to be positive using sqRT-PCR (Figs. 4J and K). Further phenotypic identification of the transgenic lines showed that compared to the wild-type (WT) control, the fruit length of the transgenic lines exhibited significant growth and a significant increase in the fruit shape index; however, the fruit width showed no pronounced variation (Figs. 4L–N). Longitudinal sections of the mature fruit mesocarp revealed larger cells in OE plants (Figs. 4O–Q), indicating that *Canq10g001705* could regulate fruit length by mediating cell expansion. These results indicate the function of *Canq10g001705* in promoting fruit length.

In the RIL population, the fruit length of the lines carrying *FL-10.1*^*AA*^ increased by approximately 14.6% compared to those carrying *FL-10.1*^*BB*^ (Fig. 4C). Subsequently, we observed an additive effect between *FL-10.1* and *FL-3.2* by comparing the fruit length of lines carrying different genotypes at these loci (Fig. 4C).

### Phylogenetic analysis of pepper IQD genes and tomato SUN genes

To reveal the phylogenetic relationships between *Canq10g001705* and other IQD/SUN genes in pepper and tomato, 33 SUN family genes in tomato were obtained through reference to a previous study (Huang et al. 2013), and 50 IQD family genes in pepper were identified through local HMMER and BLASTP. A dendrogram was constructed based on the sequences of the IQ calmodulin-binding motif. The phylogenetic tree revealed that *CaSUNs* and *SlSUNs* were classified into 12 subgroups (Figure S15). Among them, the *SlSUN* that was most homologous to *Canq10g001705* was *SISUN29*, which was classified in the XII subgroup. Thus, *Canq10g001705* was named *CaSUN29*. In tomato, *SISUN1*, *SISUN10*, and *SISUN18*, which are related to fruit shape development, have been identified and classified in subgroups X, IX, and VII, respectively (Xiao et al. 2008; Bao et al. 2023, 2024).

### Trait–loci network revealing the linkage relationships among traits

**Fig. 5:**
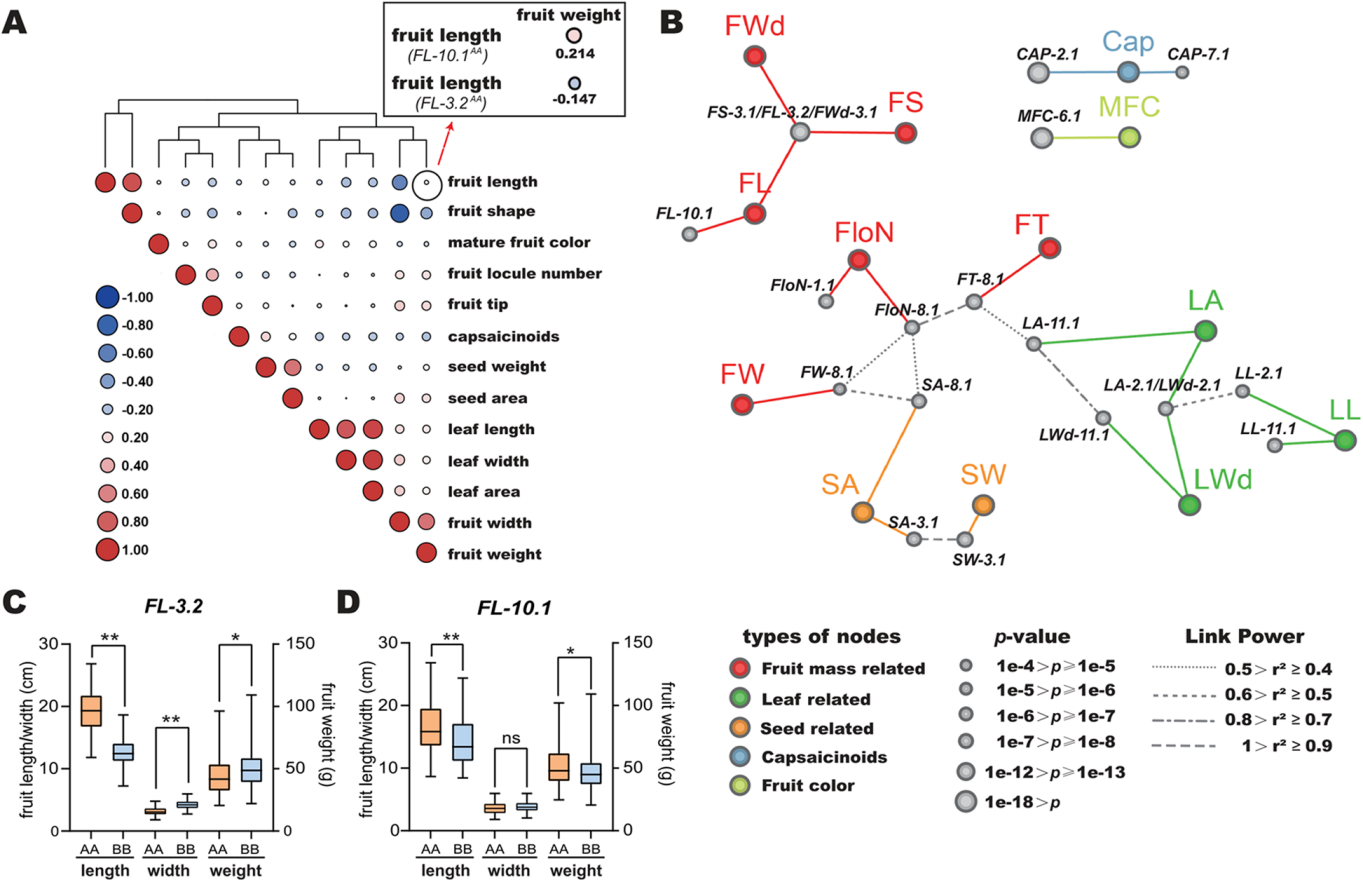
Correlation and network analysis of different traits. **A** Pearson’s correlation coefficient between traits in the RILs. The yellow square shows the correlation results for fruit length and fruit weight of the two groups of RILs, which carry either the *FL-10.1*^*AA*^ or *FL-3.2*.^*AA*^ locus. **B** Trait–loci network. Traits and loci are connected in the network if the loci were associated with the traits, and loci are connected in the network if two loci had D' > 0.8. D’, normalized disequilibrium coefficient. **C** Effects of different genotypes at the *FL-3.2* loci on fruit length, width, and weight in the RIL population. **D** The effects of different genotypes at the *FL-10.1* loci on fruit length, width, and weight in the RIL population. Student’s *t*-test was used to identify significant differences between the two groups (*, *P* < 0.05; **, *P* < 0.01; ns, not significant)

Different traits generally have a certain degree of correlation. We performed a correlation analysis of 13 agronomic traits and found that they were interrelated based on certain characteristic classifications, such as those related to fruit, leaves, and seeds. Subsequently, these traits clustered based on the varying degrees of strong or weak and positive or negative relationships among them, suggesting that some traits with highly significant correlations are genetically co-regulated (Fig. 5A). To further explore the genetic relationships among traits, a correlation network including the linkage information among loci and the association information between traits and loci was established (Fig. 5B). As expected, traits categorized together were positioned relatively closely in the network, suggesting that they had close linkage. The pungency and mature fruit color traits were relatively independent in the network, indicating that they had essentially no connection with other traits. Consistent with the pattern of trait correlations, loci controlling the associated phenotype tended to cluster into networks with greater connectivity. A notable example was the locus *FloN-8.1*, which was associated with the fruit locule number and was genetically linked to *FT-8.1*, a locus controlling the fruit tip. Compared to the AA genotype, the BB genotype of *FloN-8.1* and *FT-8.1* simultaneously and significantly increased both the fruit locule number and the proportion of concave fruits (Figure S16).

*FS-3.1/FL-3.2*, a pleiotropic locus, regulated fruit length, fruit width, and fruit shape simultaneously. In contrast, *FL-10.1* regulated fruit length independently and did not have linkage drag with other loci in the linkage network (Figs. 5B and C). Furthermore, we categorized the RILs into two groups based on whether they carried *FL-3.2*^*AA*^ or *FL-10.1*^*AA*^ and then conducted correlation analysis with the fruit weight and fruit length, respectively. In the group carrying *FL-10.1*^*AA*^, the fruit length and weight were positively correlated, and the fruit weight of lines with *FL-10.1*^*AA*^ was significantly higher than that with *FL-10.1*^*BB*^*.* Lines carrying *FL-3.2*^*AA*^ showed the opposite phenotypes (Figs. 5A and D). These findings suggest that *FL-10.1* has more flexible applications in modern breeding than *FL-3.2*.

We found that the close linkage among loci could be broken by recombination.. Within the genetic linkage network, fruit weight, fruit locule number, and seed area were pairwise connected by loci *FloN-8.1*, *FW-8.1*, and *SA-8.1*, respectively, which were closely located on chromosome 8, constituting complex local connection relationships (Figure S17A). The BB genotype of *FW-8.1* and *FloN-8.1* positively regulated the BB phenotype of fruit weight and fruit locule number, while the BB genotype of *SA-8.1* negatively regulated the BB phenotype of the seed area (Figure S17A). Therefore, when all three loci were BB genotypes, the plant had heavier fruit, along with a higher number of locules, and a smaller seed area. Several lines with the BB/BB/AA genotype in the RIL population were screened out, suggesting that genetic linkage among these three loci could be broken and recombined within the RIL population (Figure S17B). Compared to the BB/BB/BB genotype, lines with the BB/BB/AA genotype not only had heavier fruit and a higher number of locules but also had a larger seed area. Therefore, the genetic relationship between various traits and different loci was revealed through this trait–loci network.

## Discussion

RIL populations, as a type of genetically homogeneous and stable mapping population, allow researchers to replicate experiments across different environments and times. However, constructing a high-quality RIL population incurs substantial time and financial costs, and for cross-pollinated plants, such as peppers, the risk of inbreeding depression must be considered (Charlesworth and Willis 2009). In this study, the phenotypic variation and genetic basis of a stable F_10_ RIL population was investigated, and 19 significantly associated loci were identified among 13 differential traits, including 10 new loci that had not been previously identified. The successful localization of multiple known QTLs and genes and the discovery of novel loci demonstrates the robustness and effectiveness of our RIL population and mapping methodology. These findings provide a foundation for further genetic studies and breeding efforts to improve key traits in pepper. Although the major genes regulating these traits have been extensively studied, many breeding programs still struggle with uncovering minor effect loci that subtly influence fruit morphology (Barchi et al. 2009; Han et al. 2016). The advanced nature of this population allowed us to perform the high-resolution mapping of genetic loci, uncovering both well-known major effect loci, such as *FL-3.2*, *CCS*, and *CaBRX*, as well as some novel minor effect loci, such as *FL-10.1*, that regulate fruit length. This population was specifically developed to resolve these complexities in the traits of pepper fruit. Furthermore, some of these traits still have the potential for in-depth exploration to discover new loci. For example, *Cap-2.1* and *Cap-7.1* are significantly associated with capsaicinoid presence or absence, and subsequent investigations revealed that several lines carrying these loci had highly significant variations in capsaicinoid content, ranging from 42.7 to 16,584.6 µg/g FW. Consequently, we could construct F_2_ or near isogenic line populations through these selected lines to mine additional loci regulating the capsaicinoid content.

The identification and integration of minor effect QTLs have become increasingly important in modern breeding. Although major effect QTLs significantly influence key traits, they often overshadow the subtle but meaningful effects of minor QTLs (Varshney et al. 2021). However, these minor loci can play a crucial role in fine-tuning traits to achieve an optimal phenotype (Poland and Rutkoski 2016). *FS-3.1/FL-3.2* is the major locus significantly correlated with fruit length/width/shape, and a non-synonymous mutation resulting from a SNP in the coding region of candidate gene *Canq03g2458* has been reported to be associated with alterations in fruit shape (Colonna et al. 2019; Cao et al. 2022). Based on this, we constructed a new F_2_ population by selecting two RILs carrying the *FL-3.2*^*AA*^ locus but exhibiting significant differences in fruit length to fine map and clone a novel locus for fruit length, named *FL-10.1,* and with a candidate gene *CaSUN29*. The discovery of the *FL-10.1* locus demonstrates the power of our RIL population in its ability to uncover these minor effect loci that are often undetectable in conventional mapping populations. By precisely mapping and cloning *FL-10.1*, we demonstrated its role in regulating fruit length independent of width, emphasizing its significance in breeding strategies aimed at specific trait improvements. The integration of such minor effect QTLs into breeding programs offers a way to fine-tune fruit characteristics, enabling the development of pepper varieties with more tailored and desirable traits. This nuanced approach can not only improve breeding precision but also overcome the genetic bottlenecks associated with relying solely on major effect loci.

*CaSUN29* encodes a type of IQD family protein and is specifically highly expressed in fruit during the early stage of fruit development, facilitating rapid expansion of the pericarp cells during this period. IQD family proteins have been shown to be associated with fruit shape variations in multiple crops. The earliest identified was *SUN*, which regulated fruit elongation in tomato by interacting with microtubule-associated proteins (MAPs), involving microtubule reorganization (Xiao et al. 2008; Lazzaro et al. 2018). Then, IQD genes regulating fruit shape in cucumber, watermelon, and wax gourd were identified (Pan et al. 2016; Dou et al., 2018; Cheng et al. 2021). Members of the IQD family may regulate microtubule organization by interacting with calmodulin (CaM), MAPs, SPR2, and plant Rho GTPases (ROPs) or by being modulated by hormonal signals, thereby affecting the number of cells or shape (Kölling et al. 2018; Yang et al. 2020 Li et al., 2023). Unlike IQD family genes in other crops that concurrently modified both fruit length and width, such as *SlSUN*, *SlSUN10* and *SlSUN18* in tomato (Xiao et al. 2008; Bao et al. 2023, 2024), functional verification in pepper and tomato has revealed that *CaSUN29* only regulates fruit elongation, with no significant effects on fruit width. Moreover, previous studies have demonstrated that *SlSUN*, *SlSUN10*, and *SlSUN18* regulate tomato fruit shape variation by interacting with MAP proteins, which influence fruit cell arrangement and developmental processes, thereby modulating tomato fruit morphogenesis. In contrast, *CaSUN29*, acting as a specific regulator of fruit elongation, raises the critical question of whether it interacts with MAP proteins or employs distinct regulatory mechanisms. Unraveling this mechanism will be a key focus of our future research.

The primary goal of plant breeding is to integrate multiple desired traits into one variety. Nevertheless, breeders must consider trait correlations to either improve correlated traits concurrently or to mitigate undesirable effects when focusing on the improvement of a single correlated trait. The concurrent accumulation of disease resistance, high quality, and high yield traits is the perennial pursuit of breeders. Therefore, an in-depth understanding of the genetic network of different traits assists breeders in formulating an effective variety of development strategies. With continuous advancement in genomics, multiple key trait loci related to yield and quality in rice and tomato have been identified, offering distinct opportunities for breeding varieties that aggregate ideal traits from diverse sources (Jiang et al. 2012; Soyk et al. 2017; Zeng et al. 2017). However, the identification of QTLs for significant traits in pepper is insufficiently precise, and the understanding of linkage resulting in phenotypic differences among traits is not yet profound enough, resulting in considerable hindrances to the application and aggregation of complex traits in pepper breeding. In this study, a genetic network between multiple traits and the regulatory loci of pepper was constructed. Pungency and mature fruit color are two traits with relatively well-understood regulatory mechanisms whose loci were not linked to those of other traits in the network. Therefore, their selection and utilization in breeding will not have an impact on other traits. However, the loci of the majority of traits were linked to other traits to varying degrees. For example, the loci of fruit weight, fruit locule number, and seed area were linked in a pairwise manner, and the BB genotype of *SA-8.1* made a negative contribution to the BB phenotype. Therefore, in future research, using appropriate lines to generate corresponding F_2_ or near isogenic line populations to precisely map and validate these loci is necessary. Additionally, linked markers should be developed to break the linkage drag associated with the superior alleles for these traits.

Although *FL-3.2* is not linked to other loci in the network, it is a pleiotropic locus that simultaneously regulates fruit length, width, and shape. Breeders cannot utilize this locus for the precise breeding of either fruit length or width alone, and this limitation cannot be overcome through recombination or the use of molecular markers. The uniqueness of the *FL-3.2* locus leads to its current presence only in regionally distinctive Chinese pepper varieties, such as specific linear pepper cultivars (Cao et al. 2022). Therefore, this locus can be prioritized in breeding programs targeting specific linear pepper varieties; however, it may not meet the requirements for developing other pepper cultivars with specific demands on fruit width traits in breeding practices. In contrast, the specific effect of *FL-10.1* on fruit length combined with its lack of linkage to other undesirable traits makes it a versatile tool in breeding programs. It enables breeders to focus on precise fruit trait modifications without the risk of unintended alterations to other characteristics, which is often challenging with genes that have broader effects, such as *FL-3.2*. Moreover, the enhanced fruit weight observed in lines with the *FL-10.1* locus further increased its breeding value (Fig. 5D). This distinction between *FL-3.2* and *FL-10.1* highlights the importance of identifying and utilizing loci driving targeted trait improvements, leading to more refined and market-adapted pepper varieties. Thus, this RIL population represents a valuable resource for pepper genetics, providing new insights into the intricate genetic architecture of fruit morphology. By identifying loci that have minor but meaningful effects, our findings pave the way for more precise genetic improvements in pepper breeding programs, enabling the development of cultivars with optimized fruit traits to meet for diverse agricultural and market demands.

## Methods

### Plant materials and phenotyping

The RILs were derived through single-seed descent from a cross between two advanced inbred lines, BVRC1 and BVRC25, with various differential phenotypes. All inbred lines were self-pollinated by the single seed descent method to the F_10_ generation (*n* = 216). The experiment was conducted in the greenhouse of the Beijing Vegetable Research Center. Seedlings were grown in nurseries for 30–40 days before being transplanted to the field. The RILs used for phenotypic investigation were planted with six individuals per line. Thirteen agronomic traits were measured. Table S1 lists and describes the detailed information of these traits, their measurement methods, and their sample sizes. Agronomic traits were measured in two growing cycles (2021 spring/summer [SS] and 2023SS). The biological replicates for each RIL in two years were utilized for phenotypic correlation analysis (Table S3). The F_2_ population was generated through hybridization of two independent RILs.

### Capsaicinoid measurement

After extracting the placenta, the samples were pretreated according to the method described by *Cao *et al. (Cao et al. 2022). The analysis method for the filtrate of the sample was adjusted. The filtrate was injected into the ultra-high-performance liquid chromatography (UPLC) system (Waters, USA) and analyzed using an AQUITY UPLC HSS T3 1.8 μm (2.1 × 100 mm; Waters, USA) column and a photodiode detector. The mobile phase was a mixture of methanol and distilled water (volume ratio of 65:35), and the flow rate was 0.20 mL/min. The injection volume was 1 mL, and the column temperature was 30 °C. The detection wavelength was 280 nm, and the detection times for capsaicin and dihydrocapsaicin were 6.95 and 10.38 min, respectively. External standard solutions of capsaicin and dihydrocapsaicin (J&K Scientific, China) were prepared at a concentration of 1 mg/mL and diluted with methanol and tetrahydrofuran (1:1, v:v) to prepare a series of standard working solutions with concentrations of 0.8, 0.4, 0.2, 0.1, 0.05, 0.025, and 0.0125 mg/mL. The standard solutions were then measured under the liquid chromatography conditions described above. The capsaicin and dihydrocapsaicin concentrations were plotted as the vertical axis, while the corresponding peak area was plotted as the horizontal axis. The standard curve and linear regression equation were calculated. After the prepared sample solution was measured, calibration was conducted at multiple points, and quantification was carried out by means of peak area integration values. Finally, the capsaicin and dihydrocapsaicin contents were converted to ug/g FW.

### Sequencing, variant calling and annotation

Leaves were collected from all RILs and subsequently submitted to Majorbio (https://www.majorbio.com/) for DNA library construction. The prepared libraries were subjected to resequencing on their Illumina platform, generating 150 bp paired-end reads. Initial processing of the raw sequencing data was performed using Trimmomatic v0.33 to eliminate adapter sequences and produce high-quality clean data. These clean reads were then aligned to the Qiemen reference genome using BWA0.7.1 (Li and Durbin 2009; Zhang et al 2025). The resulting SAM files were converted into BAM format and sorted using SAMtools. Duplicate reads in the BAM files were marked and removed through the MarkDuplicates function implemented in GATK (version 4.1.7). Variant calling was conducted using GATK’s HaplotypeCaller with default parameters, yielding raw VCF files. Finally, functional annotation of the identified variants was performed using ANNOVAR (Wang et al. 2010).

### Recombinant bin construction

The recombination bin construction methodology was adapted from the approach described by Huang et al. (2009) with modifications. Each SNP across the 216 RILs was genotyped as AA, BB, or heterozygous. Using a sliding window approach encompassing 15 consecutive SNPs, we assigned parental genotypes when at least 9 out of 15 SNPs exhibited identical genotypic patterns (i.e., AA, BB, or heterozygous). A recombination bin was defined as a genomic region within an RIL containing consecutive SNPs sharing the same parental genotype. Genomic intervals between adjacent SNPs with discordant parental genotypes were excluded from analysis, and recombination bins shorter than 250 kb were subsequently filtered out. In cases where genotype determination was ambiguous, both alleles were annotated as "unavailable." The recombination bin map for the entire RIL population was constructed by integrating recombination bin information from individual RILs and analyzing recombination breakpoints across all lines. To evaluate the population structure, we performed principal component analysis and genotypes clustering analysis on the 216 RILs based on their recombination bin patterns. The results are visualized in Figs. 1B, S1, and S2.

### GWAS analysis

GWAS were conducted using GEMMA (Zhou and Stephens 2012). Initially, a kinship matrix was computed using GEMMA to account for population structure. Subsequently, a linear mixed model (LMM) was employed to analyze the population structure, yielding p-values for all bins across 13 traits.

### Preparation and observation of paraffin sections

Pericarps from RILs-20 and RILs-126 after 0, 5, 15, 30, and 50 d were fixed, embedded, and sectioned to a thickness of 8 μm, followed by dewaxing, as previously described (Liu et al., 2024). The sections were stained with safranin O-fast green, transparently mounted, and imaged using an Olympus light microscope. The cell length and cell area were quantified using CaseViewer software (The Digital Pathology Company). For each sample, three fields of view were examined, with three biological replicates analyzed per field.

### BSA sequencing

RILs-126 (long fruits) and RILs-20 (short fruits) from the RIL population were selected as parental lines for crossing. The fruit length trait was quantified in the F_2_ progeny derived from these crosses, and the progeny were segregated into two bulks based on their fruit lengths. Paired-end reads obtained from Illumina sequencing were processed using Trimmomatic v0.33 to eliminate low-quality reads and adapter sequences. The high-quality reads were subsequently aligned to the Qiemen reference genome using BWA v0.7.17 (Li and Durbin 2009; Zhang et al 2025); the results were output as SAM files, which were then converted to BAM files using SAMtools. Duplicate reads were removed utilizing GATK's MarkDuplicates, and SNP variant detection was conducted via GATK's HaplotypeCaller to produce raw VCF files. SNP loci within the VCF files were analyzed using the SNPindex parameter in OcBSA software (Zhang et al. 2024) to generate SNPindex plots. These plots facilitated the analysis of the genomic distribution of SNP loci across the two bulks and enabled the identification of genomic regions associated with fruit length traits.

### RNA sequencing (RNA-seq)

Pericarp tissues from the RILs-126 parental line were collected at various developmental stages, with each time point comprising three biological replicates. The collected pericarp tissues were flash-frozen in dry ice and subsequently dispatched to Majorbio (https://www.majorbio.com/) for RNA-seq library construction. A total of 15 RNA-seq libraries were prepared and subjected to sequencing on an Illumina HiSeq2000 platform. Post-sequencing, the raw 150 bp paired-end reads underwent quality filtering and were aligned to the Qiemen reference genome utilizing Hisat2 (Zhang et al 2025). The resultant SAM files were converted into BAM format and processed using SAMtools, with gene feature quantification performed via featureCounts v1.5.3 (Liao et al. 2014). Differential expression analysis between samples from distinct developmental stages was conducted using the R package DESeq2, employing fragments per kilobase of transcript per million mapped reads (FPKM) values as input. DESeq2 implements a methodology for differential expression analysis predicated on normalized count data, with p-values adjusted via the Benjamini–Hochberg method to mitigate false discovery rates (FDR). DEGs were identified based on the following criteria: FPKM > 1, a fold-change > 1.5 or < 0.67 in at least one tissue, and an adjusted p-value (padj) < 0.05. Subsequently, GO term and KEGG pathway enrichment analyses of the DEGs were executed using the DAVID database (https://david.ncifcrf.gov/), with the closest homologous genes from Arabidopsis thaliana serving as references.

### Virus-induced gene silencing

The function of the *Canq10g001705* gene was investigated using the VIGS method. The 300-bp fragment most suitable for silencing in the *Canq10g001705* coding sequence was analyzed on the SGN website (https://vigs.solgenomics.net/) and inserted into the TRV2 vector. TRV1, TRV2, TRV2::*PDS*, and TRV2::*Canq10g001705* were transformed into *A. tumefaciens* strain GV3101. The mixture was used to infect “RILs-126” at the cotyledon expansion stage using the vacuum infiltration method (0.1 MPa for 8 min). A mixture of TRV1 and TRV2::*Canq10g001705* was used to silence *Canq10g001705*. A mixture of TRV1 and TRV2 served as the negative control, and a mixture of TRV1 and TRV2::*PDS* was regarded as the positive control. Seedlings were transplanted after being cultured in the dark at 22 °C for 48 h. The expression levels were detected, and the fruit length was measured after 30 d of fruit development.

### Transformation of tomato plants

The coding sequence of *Canq10g001705* was inserted into the overexpression vector Super1300-GFP using the ClonExpress Ultra One Step Cloning Kit (Vazyme, China). Tomato (Solanum lycopersicum “Ailsa Craig”) transformation was performed following a previously described protocol with slight modifications (Sun et al. 2006). Specifically, upon the establishment of robust roots, the transgenic plants were transplanted into soil and co-cultivated in a greenhouse alongside non-transformed control tomato plants. Positive transformants were identified through PCR amplification using primers specific to the vector sequence and the target gene. Expression levels of the transgene were assessed via sqRT-PCR. Fruits harvested from the T1 generation of two independent transgenic lines were subjected to phenotypic analysis.

### Quantitative reverse transcription PCR (qRT-PCR) analysis

Total RNA was extracted utilizing a FastPure Universal Plant Total RNA Isolation Kit (Vazyme, China), adhering strictly to the manufacturer's guidelines. Following extraction, RNA from each sample was subjected to reverse transcription, employing the HiScript IV RT SuperMix for qRT-PCR with gDNA wiper (Vazyme, China). qRT-PCR was conducted on LightCycler 480 Real-Time PCR System (Roche, Switzerland) using the ChamQ Universal SYBR qPCR Master Mix (Vazyme, China) under optimized thermal cycling conditions: an initial denaturation at 95 °C for 5 min, followed by 40 cycles of denaturation at 95 °C for 10 s, annealing at 60 °C for 10 s, and extension at 72 °C for 30 s. The *ubiquitin* gene (*UBI*) served as an internal control to normalize gene expression levels. All of the primer sequences utilized in this study are comprehensively detailed in Table S8.

### Identification of SUN/IQD genes in pepper and tomato

To identify members of the IQD gene family in pepper, the hidden Markov model (HMM) profile of the IQ calmodulin-binding motif (PFAM: PF00612) was retrieved from the PFAM database (http://pfam.xfam.org/) and 33 previously characterized IQD protein sequences from tomato (*Solanum lycopersicum*) were used as reference(Huang et al. 2013). A combined approach employing HMMER (v3.3.2) and BLASTP was implemented to screen homologous proteins in the Qiemen pepper genome database (E-value threshold < 1e-5)(Zhang et al. 2025). After the removal of redundant sequences, the retained sequences were subjected to conserved domain identification using the NCBI conserved domain database (CDD; https://www.ncbi.nlm.nih.gov/cdd). Sequences harboring a complete IQ calmodulin-binding motif were selected as putative members of the pepper IQD gene family. A phylogenetic tree was constructed using TBtools (Chen et al. 2020), and the resultant phylogenetic relationships were visualized using the Interactive Tree of Life platform (Letunic and Bork 2024).

### Construction of the trait–loci network

The network connectivity between traits followed the method described by Zhang et al. (2018). The following steps were used: (i) Given traits A and B, the set of loci directly related to trait A were labelled as L(A), and the set of loci directly related to trait B were labelled as L(B); (ii) the set of loci directly connected to trait A or connected to trait B via other loci was labelled as C(A, B). The set of loci directly connected to trait B or connected to trait A via other loci was labelled as C(B, A); (iii) for each locus directly connected to trait A, the rounded logarithm of the *P*-value of GAPIT was labelled as* P*(i, A); (iv) The network connectivity between traits A and B was calculated as follows: $\text{NC}\left(\text{A},\text{ B}\right)=\sqrt{\frac{\sum \text{i}\in \text{C}{\left(\text{A},\text{ B}\right)}^{\text{P}\left(\text{i},\text{ A}\right)}}{\sum \text{i}\in \text{C}{\left(\text{A}\right)}^{\text{P}\left(\text{i},\text{A}\right)}}\times \frac{\sum \text{i}\in \text{C}{\left(\text{B},\text{A}\right)}^{\text{P}\left(\text{i},\text{ B}\right)}}{\sum \text{i}\in \text{C}{\left(\text{B}\right)}^{\text{P}\left(\text{i},\text{B}\right)}}}$

Pairwise r^2^ values were calculated between all significant bins using the 2023SS phenotypic data in PLINK. The construction of the trait–loci network followed the method described by Zhang et al. (2018). Traits and their corresponding nodes were regarded as nodes, and trait nodes were colored based on different categories. The size of the locus nodes was determined by their lowest *P* values. The solid line represents the connection edges between the trait and the locus. If D’ > 0.8 for linkage disequilibrium (LD), the two loci were connected by different dashed lines representing the connection edges, based on the value of r^2^. The network graph was generated using CYTOSCAPE 3.10.2 (Shannon et al. 2003). The node positions were determined using the edge-weighted spring-embedded layout, and all nodes had default parameters.

## Supplementary Information

Supplementary Material 1: Table S1. Descriptions of 13 agronomic traits and measurement methods. Table S2. Phenotypic differences in all traits between the two parents(BVRC1 and BVRC25). Table S3. Pearson’s correlation coefficients between the replicates of each trait in a single season and those between the two seasons. Table S4. Re-sequencing data and average sequencing depth of parents and RILs. Table S5. Number of SNPs in each RIL. Table S6. RILs recombination frequency. Table S7. List of loci identified in RILs. Table S8. Primers utilized in this study.

Supplementary Material 2: Figure S1. Distribution of bins and genes. Figure S2. Principal component analysis (PCA) of 216 RILs by genotype information. Figure S3. Manhattan plots and quantile–quantile plots of all traits in RIL association analysis. Figure S4. Mapping of reads to the genomic region surrounding the *Canq06g000674* (*CCS*) gene in BVRC1 (red fruit) and BVRC25 (yellow fruit). The region covered with no reads in BVRC25 indicates a large sequence deletion. Figure S5. Identification of loci of capsaicinoids. Figure S6. Manhattan plot of the fruit locule number and the phenotypic variances of RILs with different genotypes at each locus. Figure S7. Manhattan plot of the fruit length, fruit width, and fruit shape. The phenotypic variances of RILs with different genotypes at each locus. Figure S8. Phenotypic variance of loci associated with seed area. Figure S9. The result of clustering the RIL lines based on their genotypes. RILs-20 and RILs-126, marked in yellow, were used to construct the fruit length mapping population. Figure S10. Analysis of sequence variations in the coding regions of *Canq10g001702*, *Canq10g001703*, and *Canq10g001704* between RILs-20 and RILs-126. Figure S11. (A) and (B) denote differences in the coding sequence and amino acid sequence between RILs-20 and RILs-126. Figure S12. RNA-seq analysis of the fruit (ovary) at different developmental stages in RILs-126. Figure S13. Analysis of the expression levels of genes within the* FL-10.1* interval in RILs-126 fruit (ovary) at different developmental stages. Figure S14. Expression level of *Canq10g001705* in different tissues. Figure S15. Analysis of the phylogenetic tree of *Canq10g001705* (*CaSUN29*) and other pepper IQD genes and tomato SUN genes. Figure S16. Phenotypic variance of linked loci for fruit tip and fruit locule number. Figure S17. Linkage and recombination among the loci based on the fruit weight, fruit locule number, and seed area.

## Data Availability

The data supporting the results of this article are included within the article and its additional files. The raw data from the BSA-seq (CRA023250) and RNA-seq (CRA022918) samples are available at the National Genomics Data Center(NGDC).

## Abbreviations

BRX: Brevis Radix
Cap: Capsaicinoids
CCS: Capsanthin/Capsorubin synthase
FDR: False discovery rate
FL: Fruit length
FloN: Fruit locule number
FPKM: Fragments per kilobase of transcript per million mapped reads
FS: Fruit shape
FT: Fruit tip
FW: Fruit weight
FWd: Fruit width
GWAS: Genome-wide association study
HSD: Honestly significant difference
InDel: Insertion/Deletion
IQD: IQ67 domain
KEGG: Kyoto Encyclopedia of Genes and Genomes
LA: Leaf area
LD: Linkage disequilibrium
LL: Leaf length
LMM: Linear mixed model
LWd: Leaf width
MFC: Mature fruit color
OE: Overexpression
OFP: Ovate family protein
PCA: Principal component analysis
PDS: Phytoene desaturase
qRT-PCR: Quantitative reverse transcription polymerase chain reaction
QTL: Quantitative trait locus
RIL: Recombinant inbred line
SA: Seed area
SNP: Single nucleotide polymorphism
SS: Spring/Summer
SW: Seed weight
TRM: TONNEAU 1 recruiting motif
UBI: Ubiquitin
UPLC: Ultra-performance liquid chromatography
VIGS: Virus-induced gene silencing
WT: Wild type
