# Supplementary material for: Identification of major loci governing 13 agronomic traits and the fine-mapping of CaSUN29 regulating fruit length in pepper
Source: Mol Hortic. 2025 Dec 1;5:64. doi: 10.1186/s43897-025-00179-7 (PMC12667057; doi:10.1186/s43897-025-00179-7)
Supplement: Supplementary file 2 — Supplementary Material 2: Figure S1. Distribution of bins and genes. Figure S2. Principal component analysis (PCA) of 216 RILs by genotype information. Figure S3. Manhattan plots and quantile–quantile plots of all traits in RIL association analysis. Figure S4. Mapping of reads to the genomic region surrounding the Canq06g000674 (CCS) gene in BVRC1 (red fruit) and BVRC25 (yellow fruit). The region covered with no reads in BVRC25 indicates a large sequence deletion. Figure S5. Identification of loci of capsaicinoids. Figure S6. Manhattan plot of the fruit locule number and the phenotypic variances of RILs with different genotypes at each locus. Figure S7. Manhattan plot of the fruit length, fruit width, and fruit shape. The phenotypic variances of RILs with different genotypes at each locus. Figure S8. Phenotypic variance of loci associated with seed area. Figure S9. The result of clustering the RIL lines based on their genotypes. RILs-20 and RILs-126, marked in yellow, were used to construct the fruit length mapping population. Figure S10. Analysis of sequence variations in the coding regions of Canq10g001702, Canq10g001703, and Canq10g001704 between RILs-20 and RILs-126. Figure S11. (A) and (B) denote differences in the coding sequence and amino acid sequence between RILs-20 and RILs-126. Figure S12. RNA-seq analysis of the fruit (ovary) at different developmental stages in RILs-126. Figure S13. Analysis of the expression levels of genes within the FL-10.1 interval in RILs-126 fruit (ovary) at different developmental stages. Figure S14. Expression level of Canq10g001705 in different tissues. Figure S15. Analysis of the phylogenetic tree of Canq10g001705 (CaSUN29) and other pepper IQD genes and tomato SUN genes. Figure S16. Phenotypic variance of linked loci for fruit tip and fruit locule number. Figure S17. Linkage and recombination among the loci based on the fruit weight, fruit locule number, and seed area. [file 43897_2025_179_MOESM2_ESM.docx]

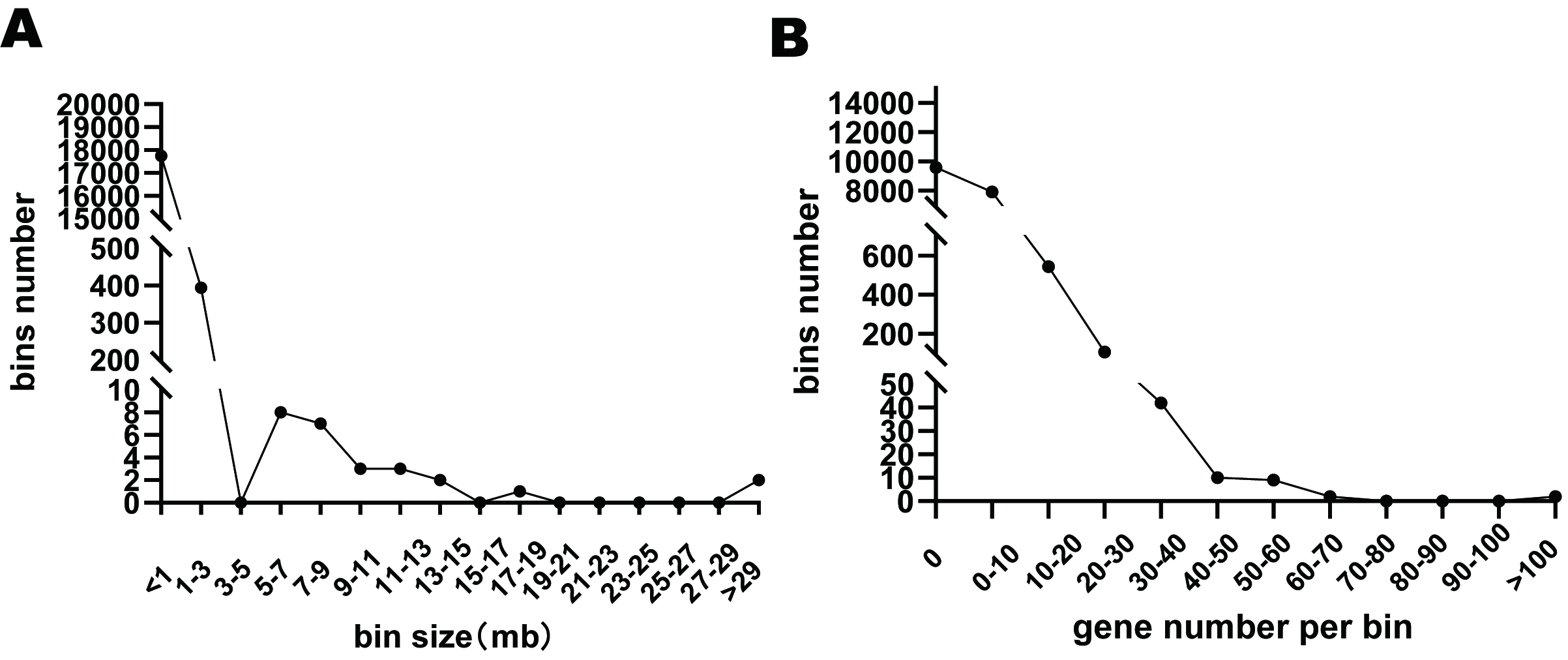


**Figure S1.** **Distribution of bins and genes**

(A) Distribution of the bin size for RILs. (B) Distribution of the number of genes per bin for RILs.


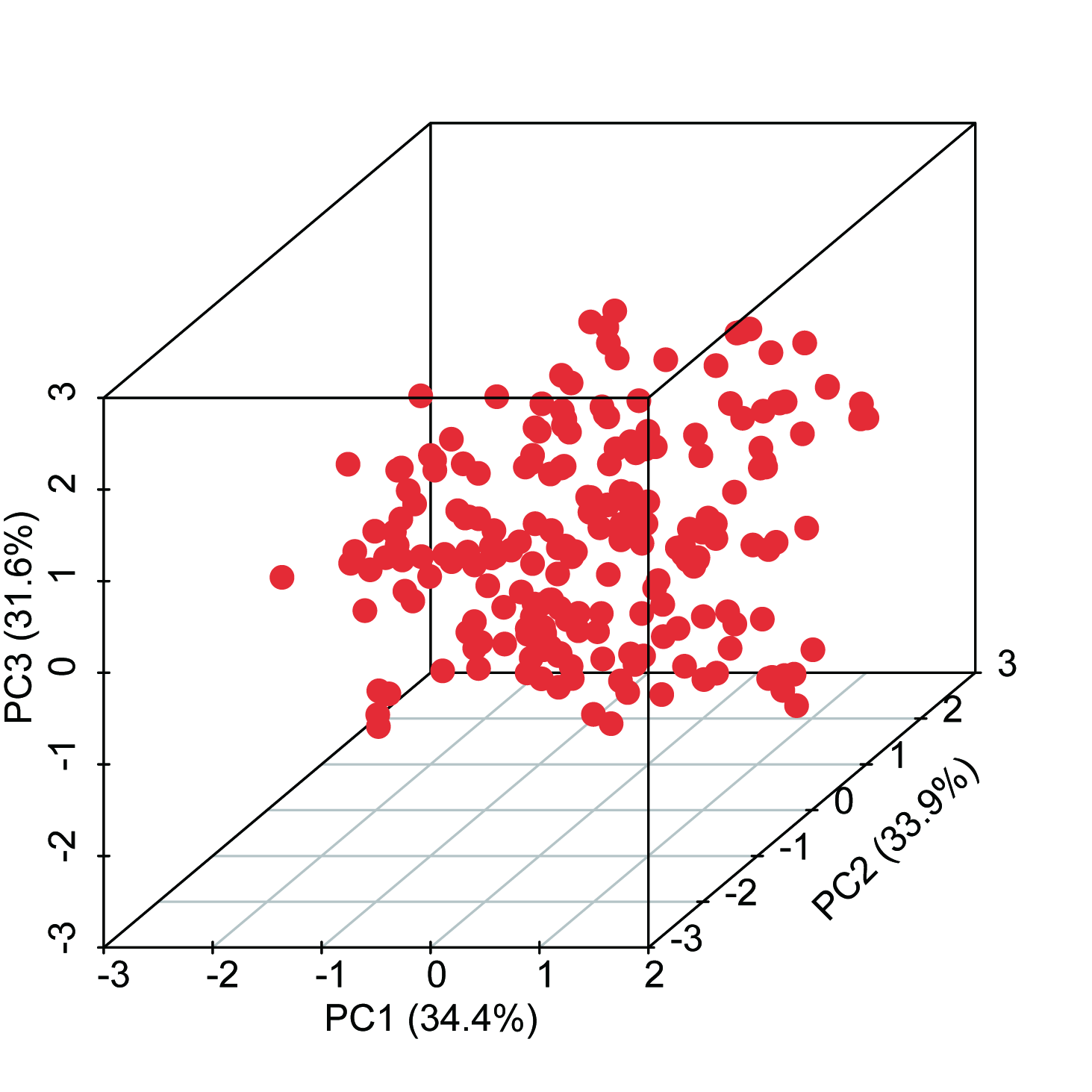


**Figure S2. Principal component analysis (PCA) of 216 RILs by genotype information.**


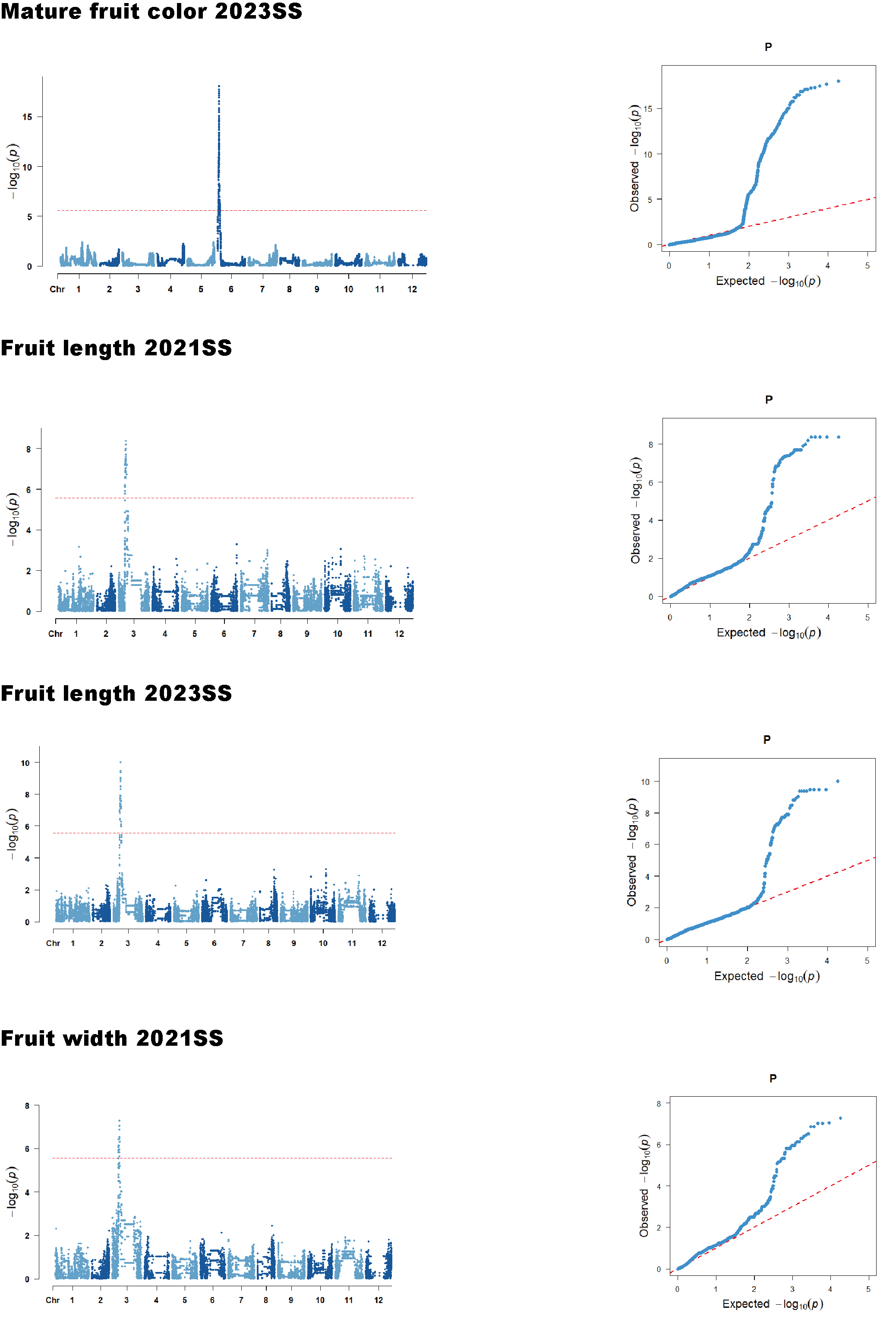


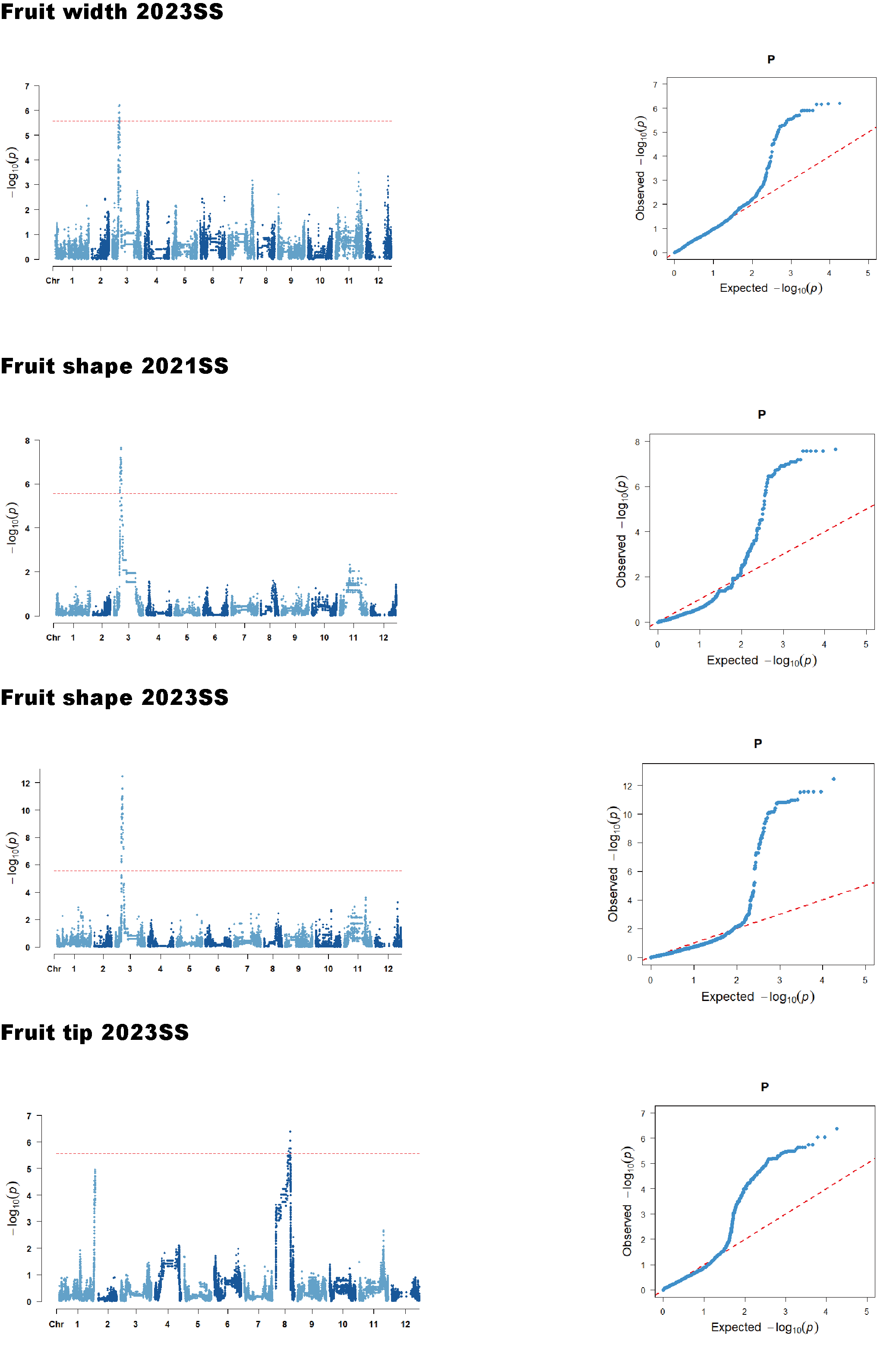


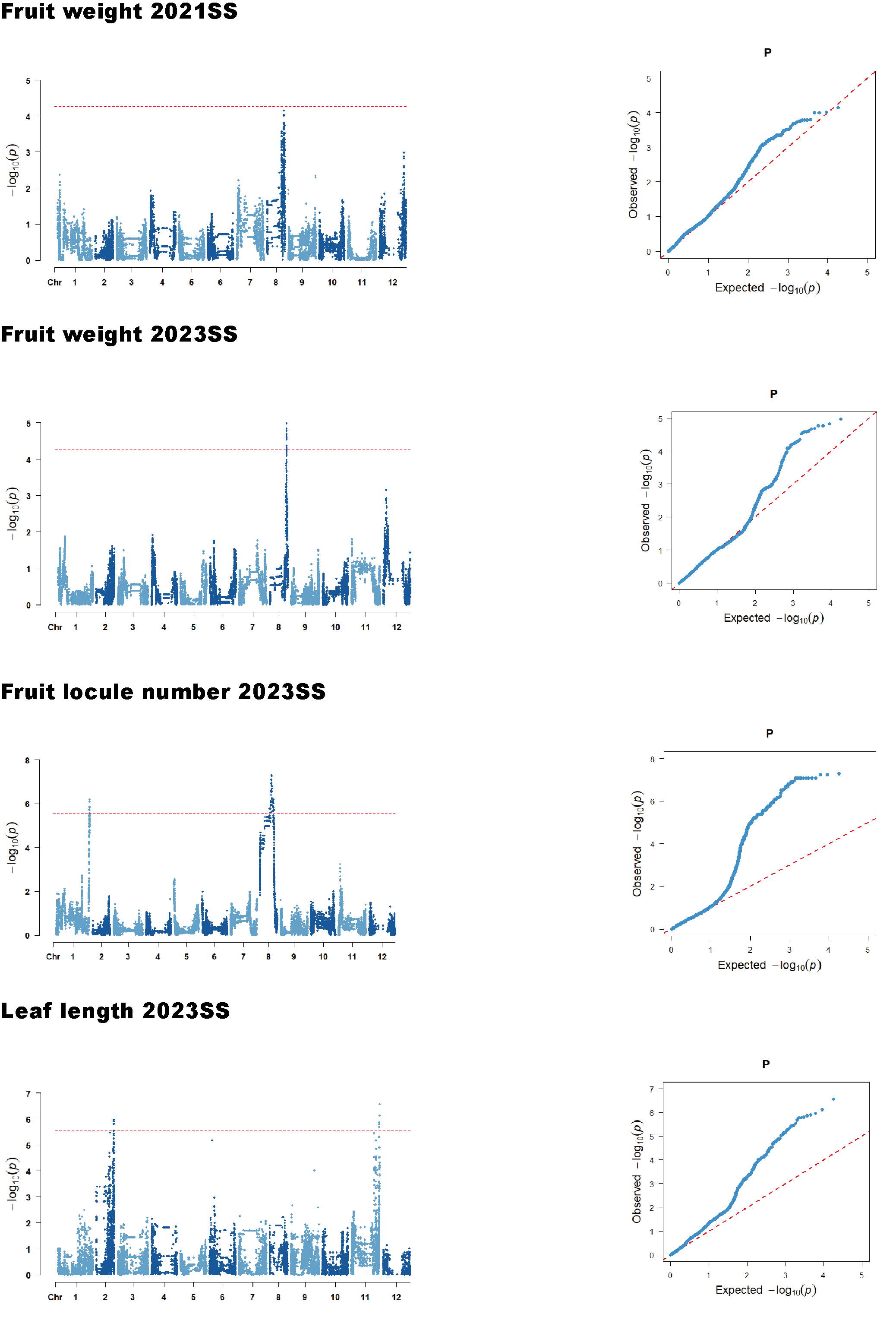


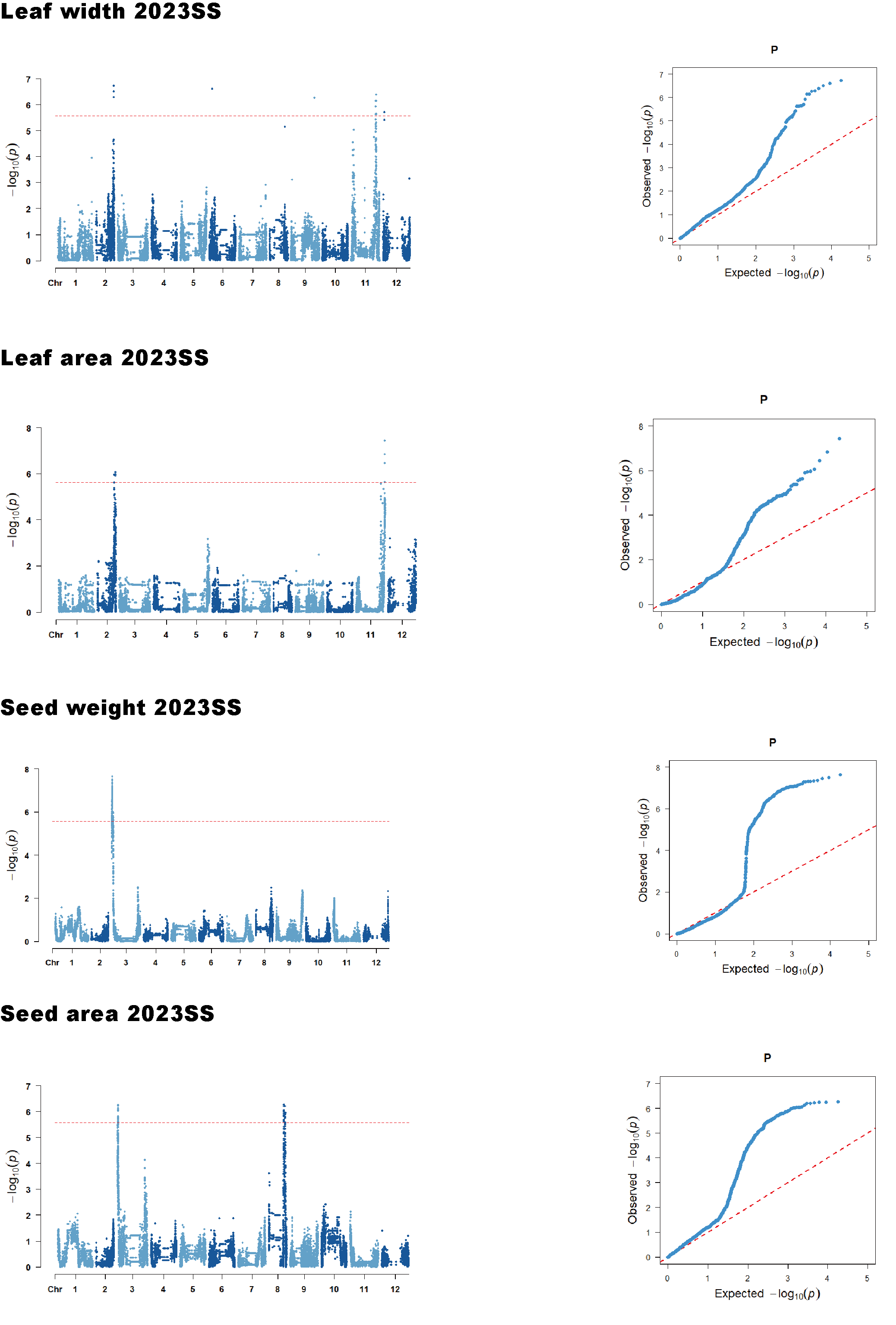


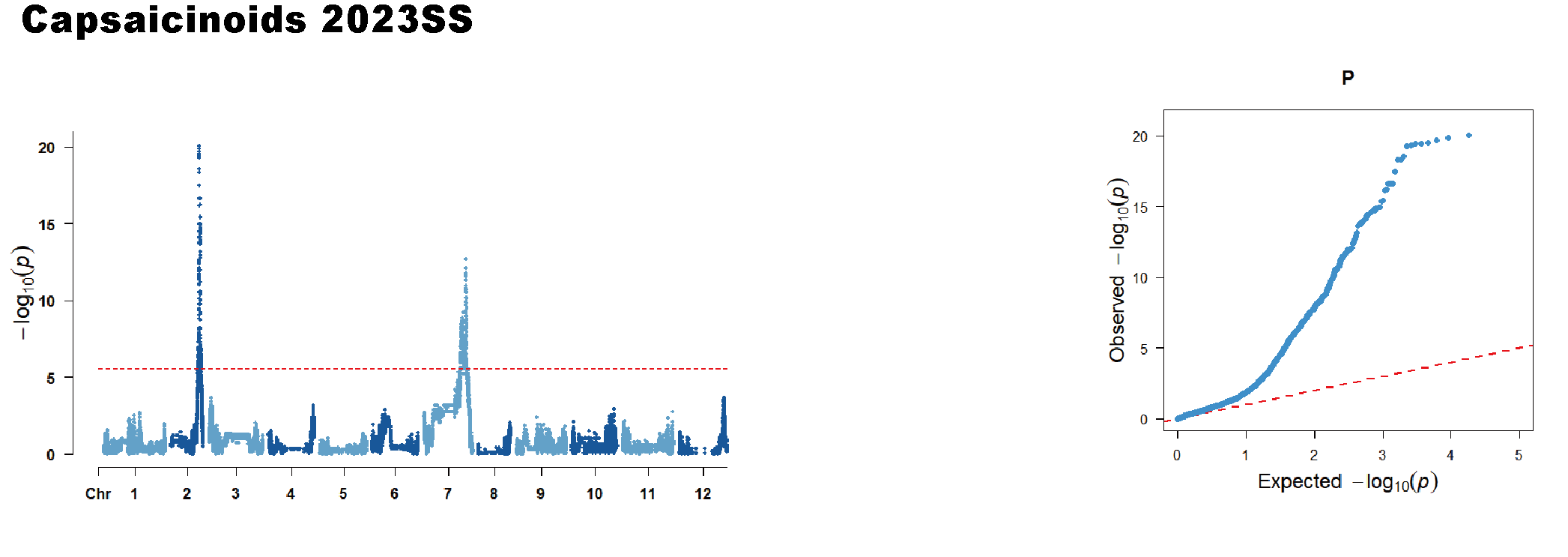


**Figure S3. Manhattan plots and quantile–quantile plots of all traits in RIL association analysis.**

**
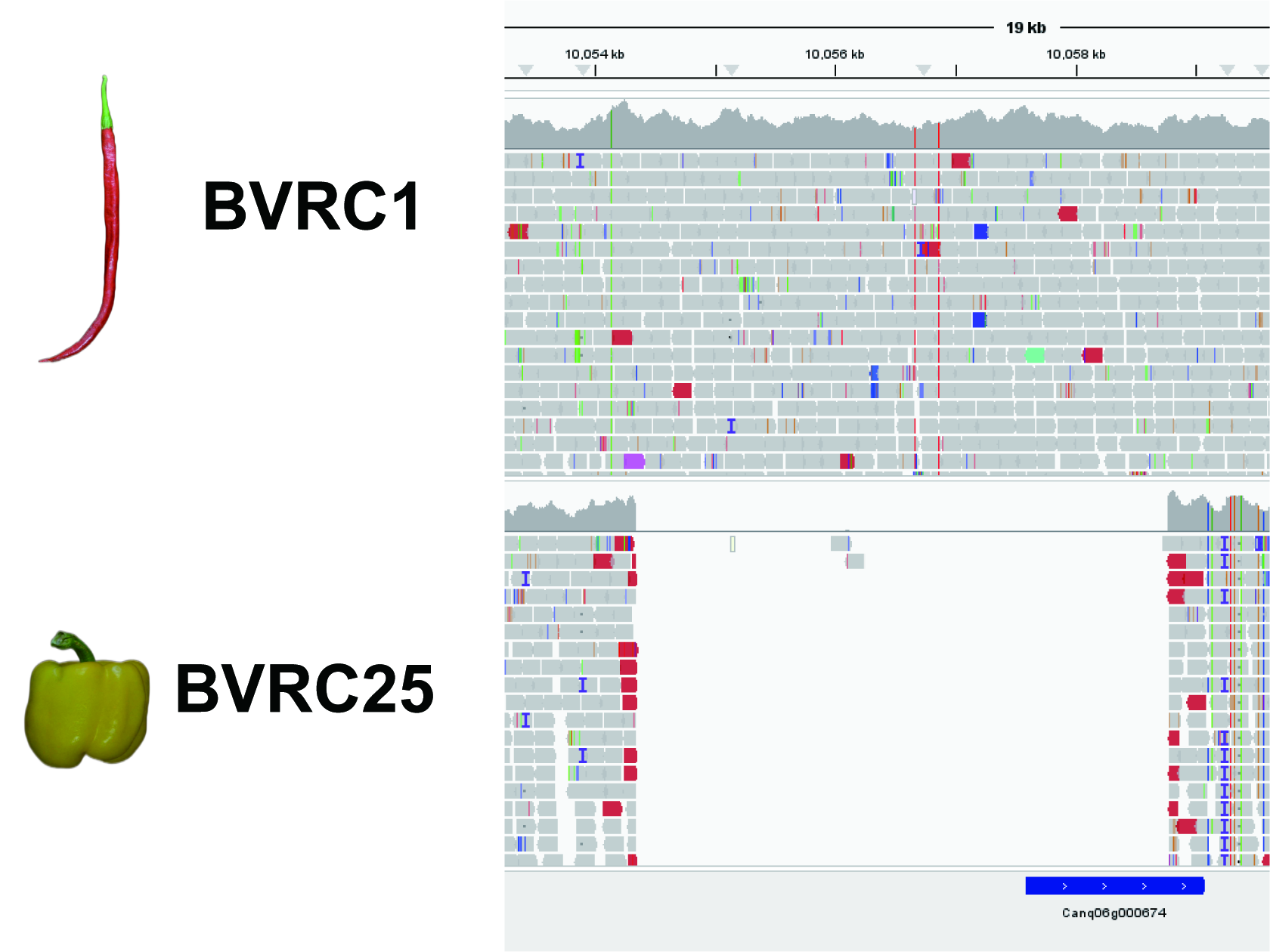
**

**Figure S4. Mapping of reads to the genomic region surrounding the *Canq06g000674* (*CCS*) gene in BVRC1 (red fruit) and BVRC25 (yellow fruit). The region covered with no reads in BVRC25 indicates a large sequence deletion.**

**
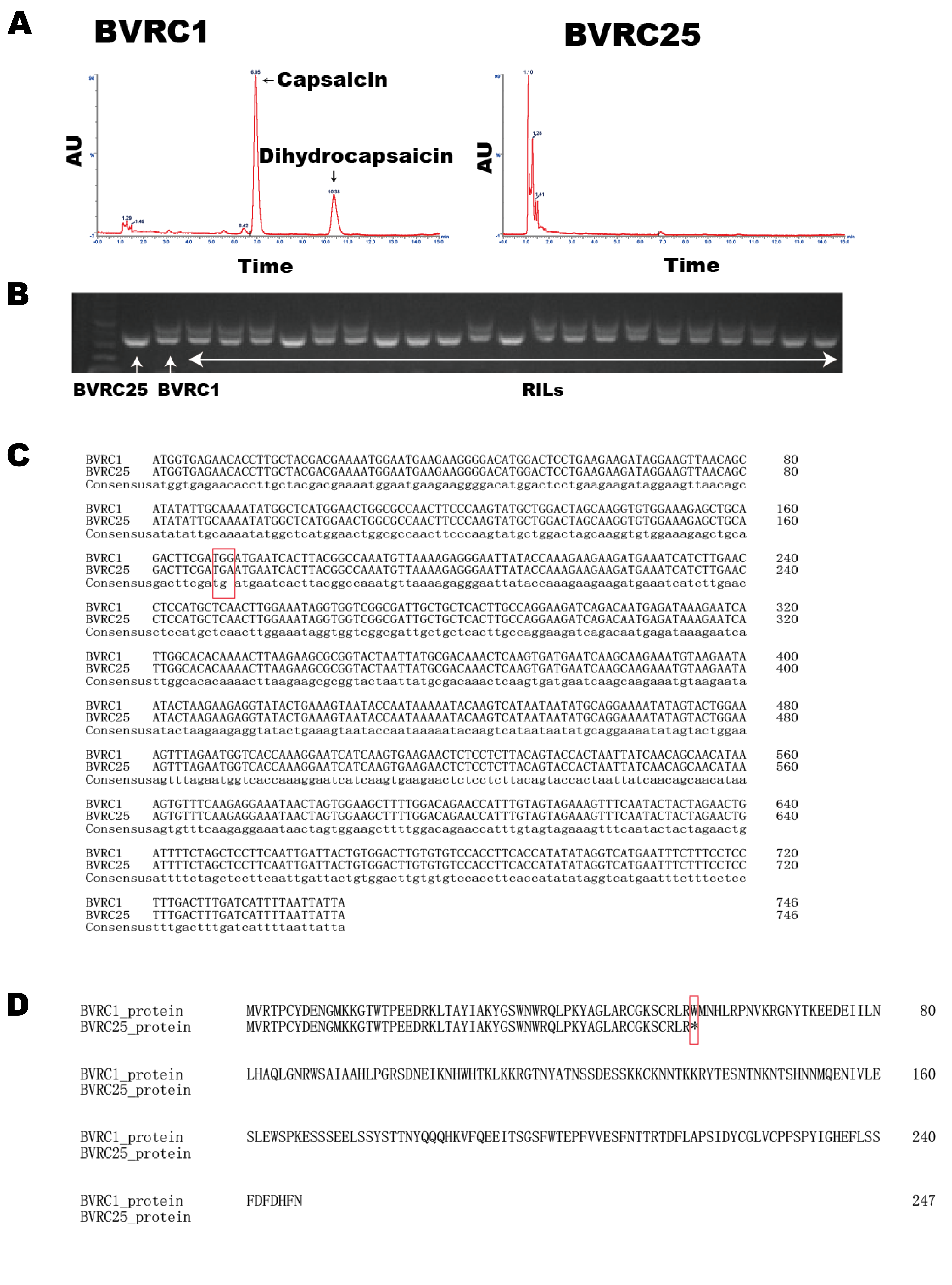
**

**Figure S5. Identification of loci of capsaicinoids.**

(A) Detection of capsaicin and dihydrocapscaicin using UPLC analyses of placenta from BVRC1 and BVRC25 at the mature fruit stage. AU, arbitrary units. (B) Detection of *Pun1* in parental lines and RILs. (C) Sequence variation of *Pun3* in the coding regions between the two parental lines. (D) Amino acid differences in *Pun3* between the two parental lines. The red-framed part is the differential region.

**
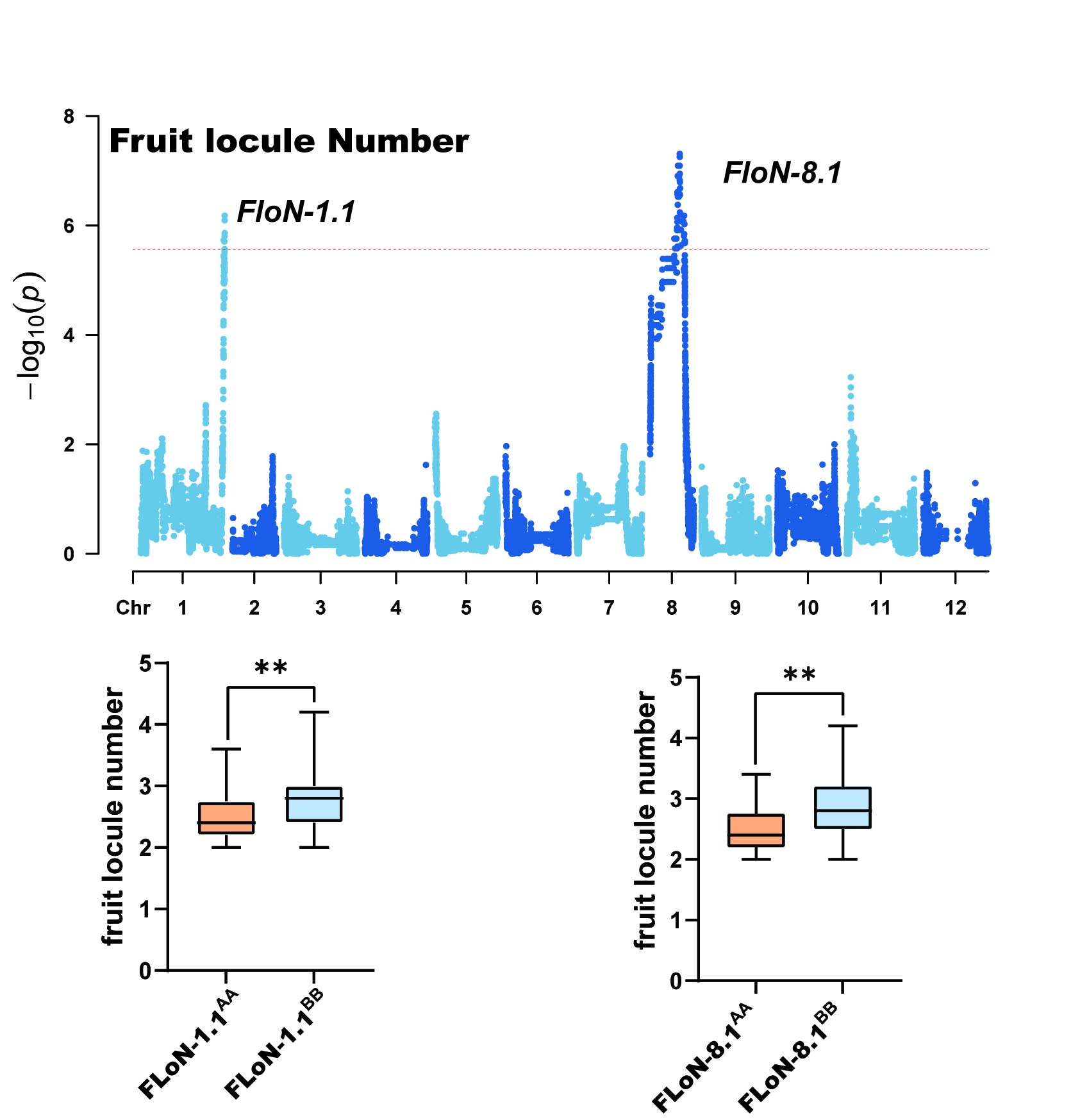
**

**Figure S6. Manhattan plot of the fruit locule number and the phenotypic variances of RILs with different genotypes at each locus.**

AA: BVRC1 genotype. BB: BVRC25 genotype.

**
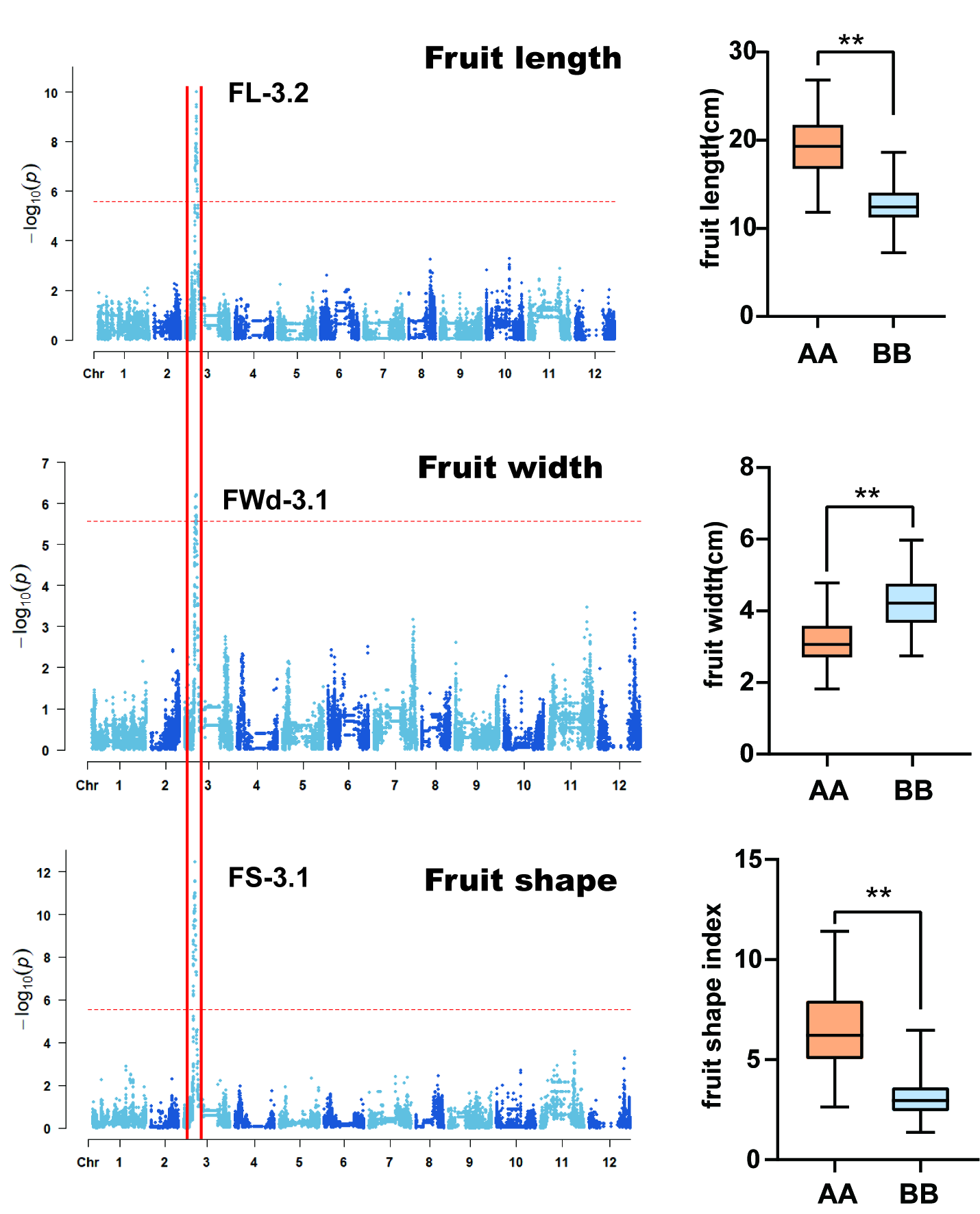
**

**Figure S7. Manhattan plot of the fruit length, fruit width, and fruit shape. The phenotypic variances of RILs with different genotypes at each locus.**

AA: BVRC1 genotype. BB: BVRC25 genotype.

*
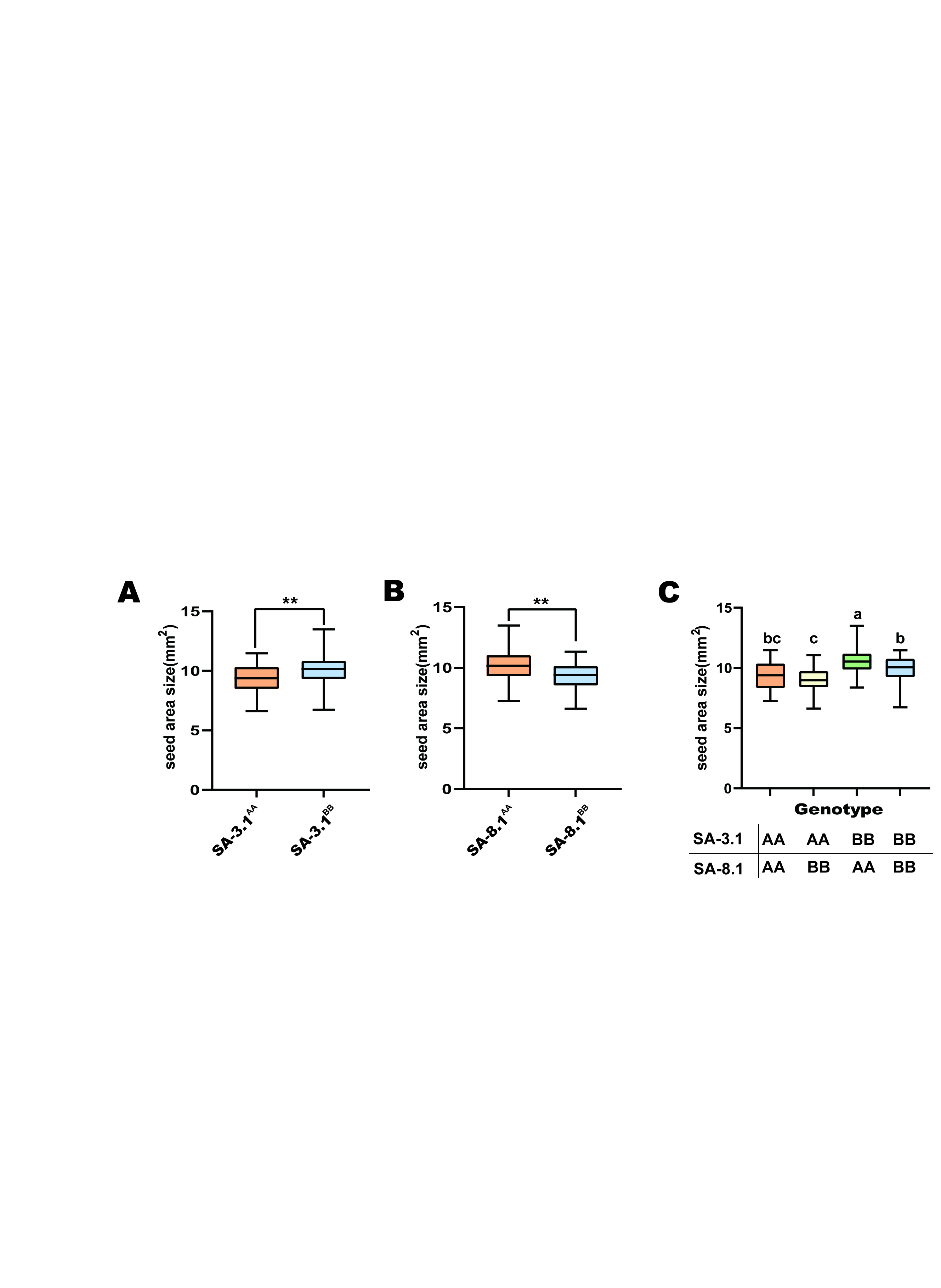
*

**Figure S8.** **Phenotypic variance of loci associated with seed area.**

(A) Phenotypic variance of locus *SA-3.1* to the seed area. (B) Phenotypic variance of locus *SA-8.1* to the seed area. (C) Combinatorial effects of *SA-3.1* and *SA-8.1* to the seed area**.** Student’s *t* test was used to identify significant differences between the two groups (**, *P* < 0.01). Tukey’s honestly significant difference (HSD) test was used to identify significant differences among multiple groups, and different letters above the boxes indicate statistically significant differences, *P* < 0.05. AA: BVRC1 genotype. BB: BVRC25 genotype.


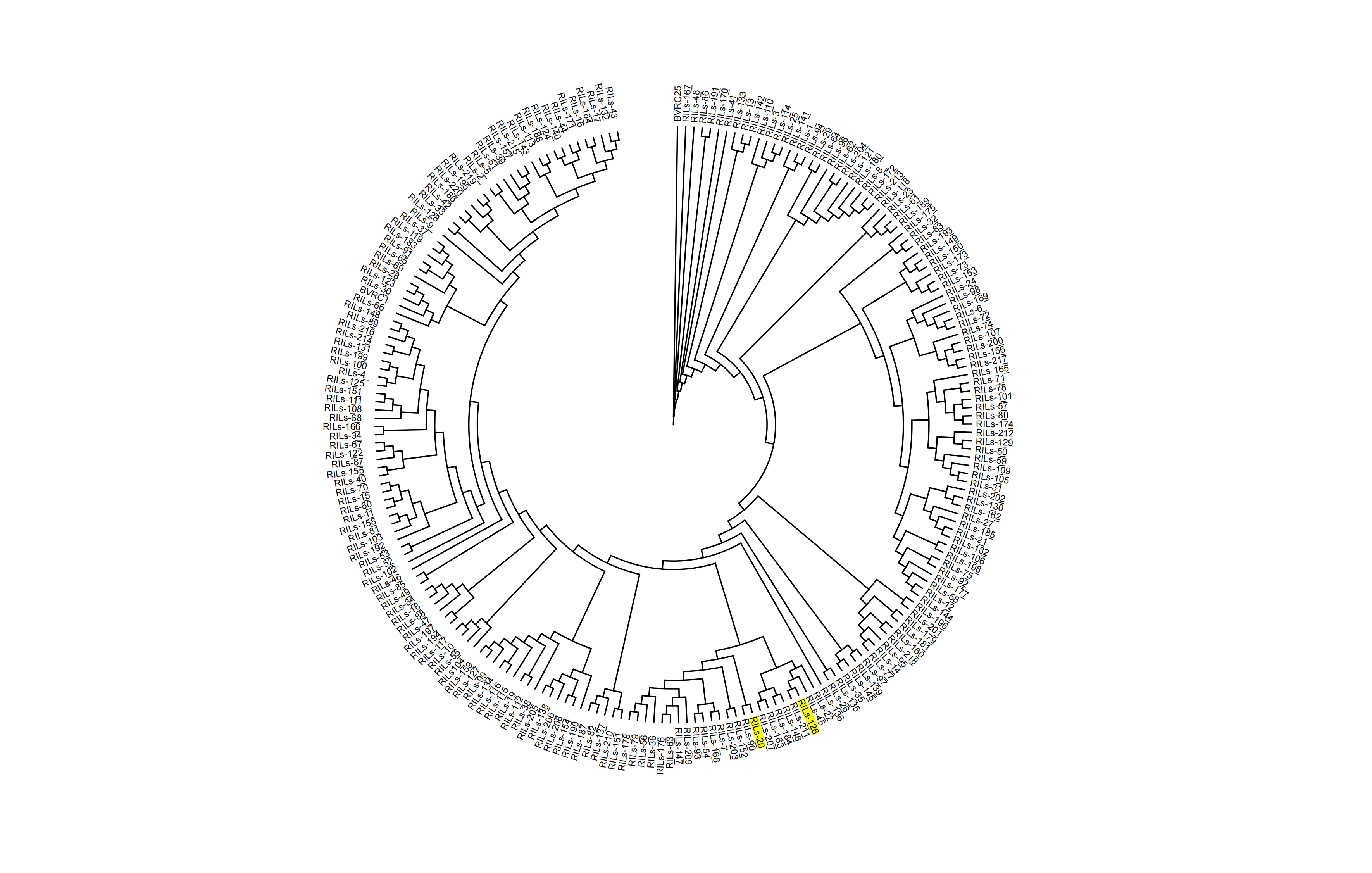


**Figure S9. The result of clustering the RIL lines based on their genotypes. RILs-20 and RILs-126, marked in yellow, were used to construct the fruit length mapping population.**


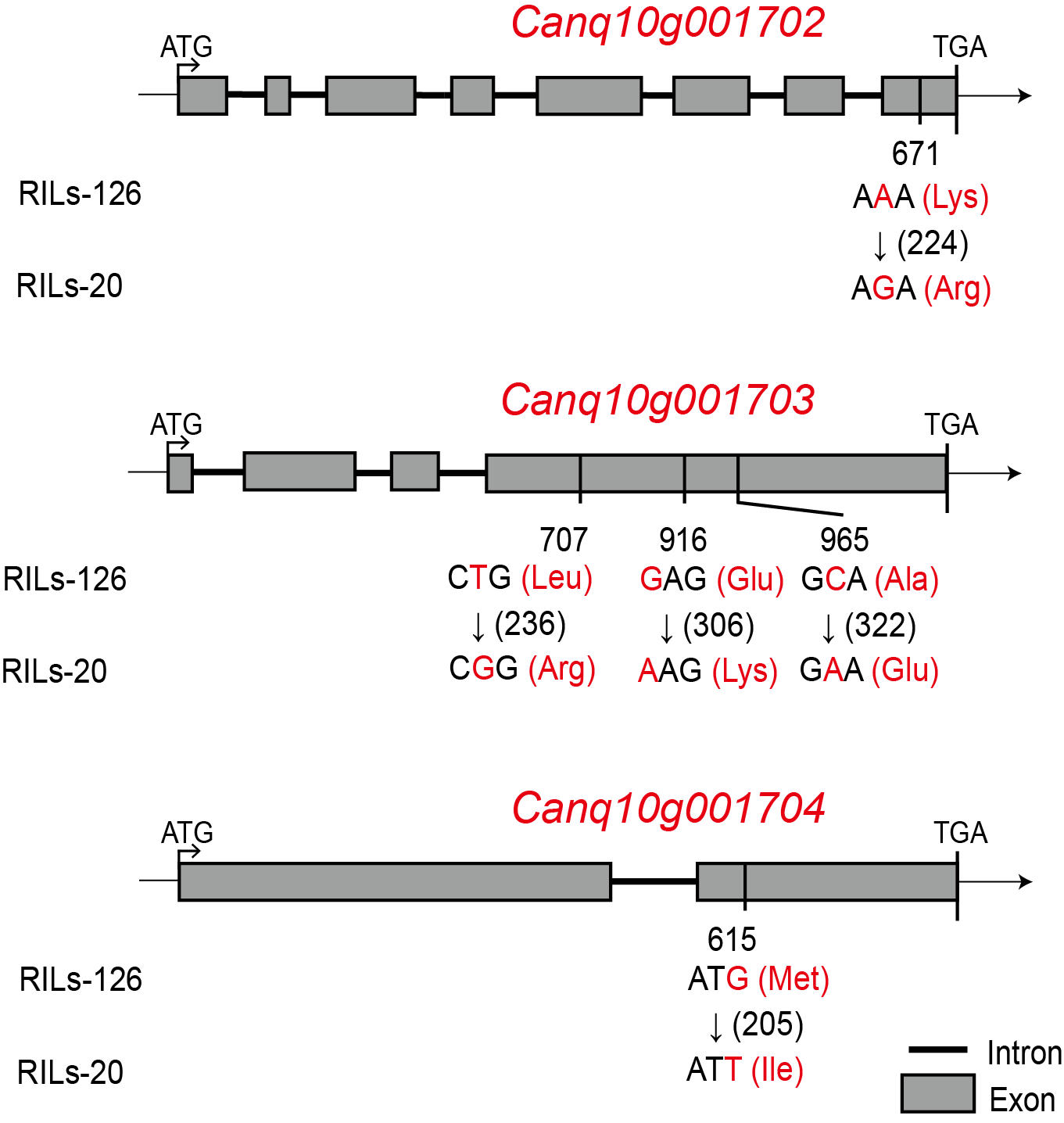


**Figure S10. Analysis of sequence variations in the coding regions of *Canq10g001702*, *Canq10g001703*, and *Canq10g001704* between RILs-20 and RILs-126.**

**
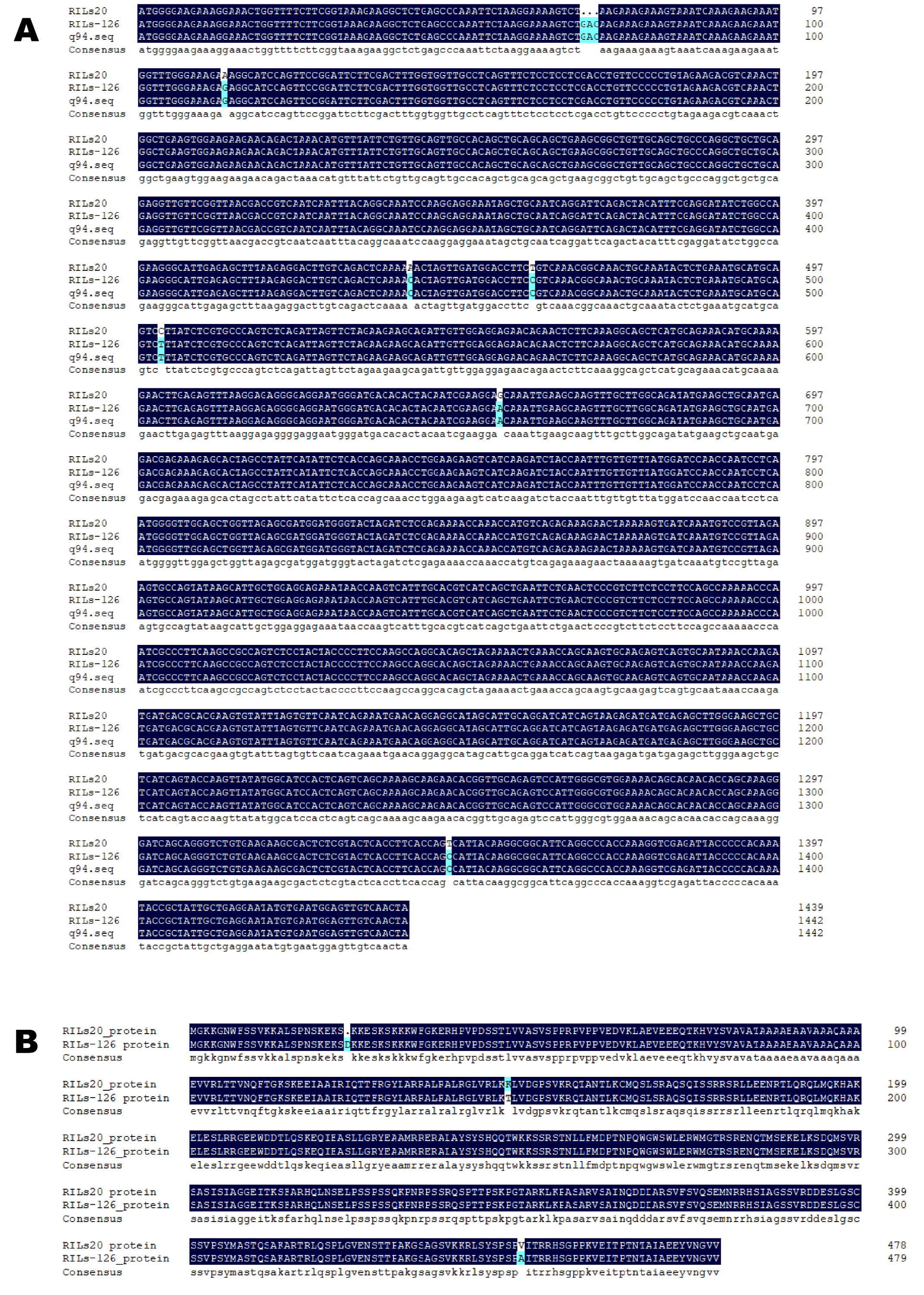
**

**Figure S11. (A) and (B) denote differences in the coding sequence and amino acid sequence of *Canq10g001705* between RILs-20 and RILs-126.**

**
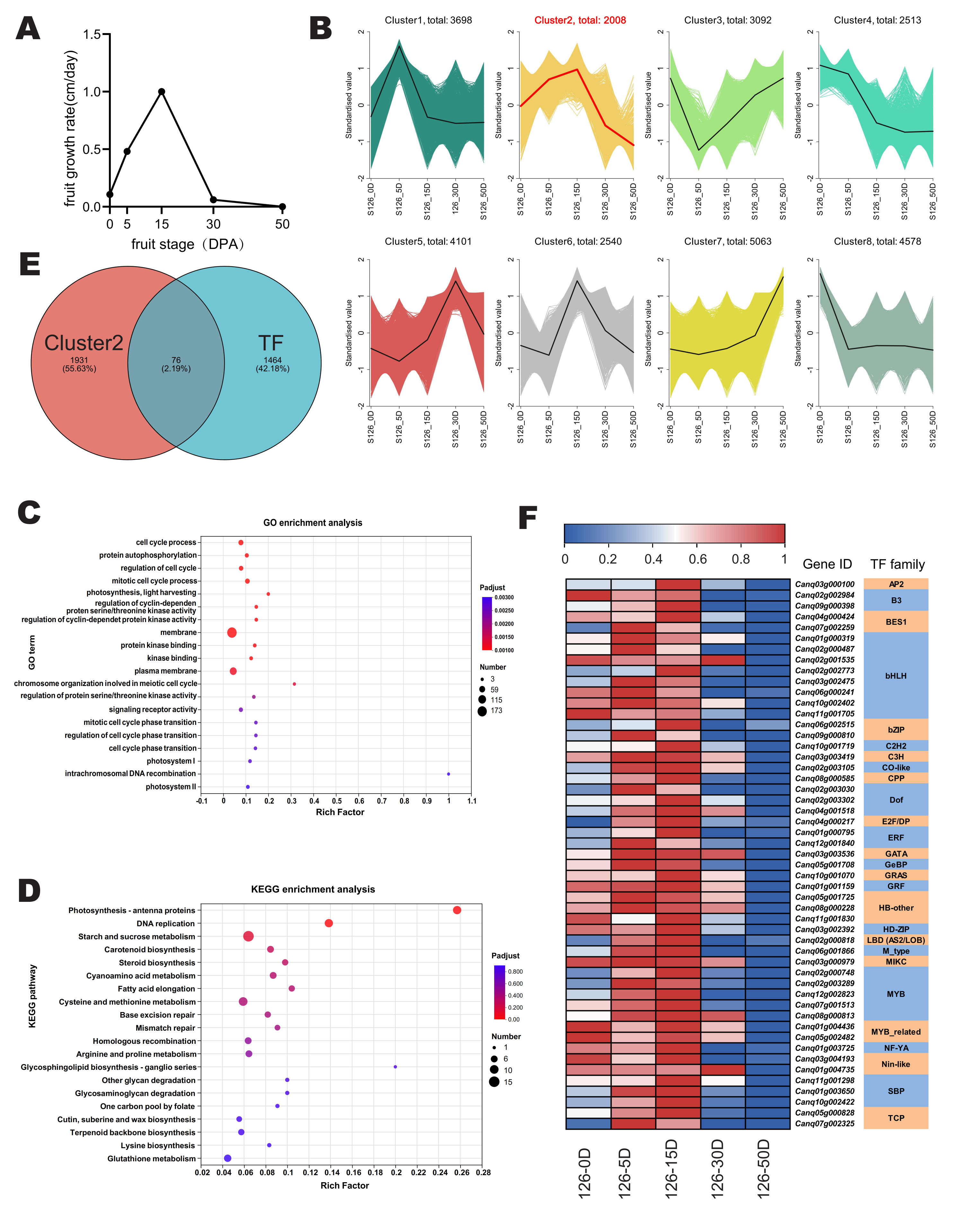
**

**Figure S12. RNA-seq analysis of the fruit (ovary) at different developmental stages in RILs-126.**

(A) Growth rate of fruit (ovary) at different developmental stages in RILs-126. (B) K-means clustering analysis of gene expression data and fruit growth rate. (C) GO enrichment analysis in Cluster 2. (D) KEGG enrichment analysis in Cluster 2. (E) The number of transcription factors in Cluster 2. (F) The 51 transcription factors in Cluster 2 and the TF families to which they belong. The genes that were almost not expressed in the fruit (average FPKM < 2) were filtered out. The expression level was normalized per row using Min–Max.


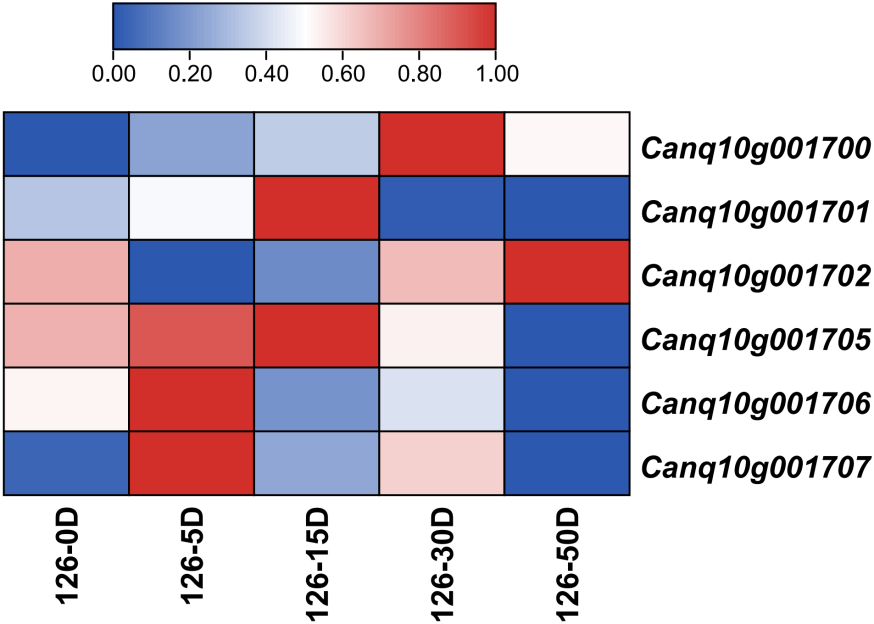


**Figure S13. Analysis of the expression levels of genes within the *FL-10.1* interval in RILs-126 fruit (ovary) at different developmental stages**

The expression level was normalized per row using Min–Max. *Canq10g001698*, *Canq10g001699*, *Canq10g001703*, and *Canq10g001704* were not expressed in the fruit (ovary).

**
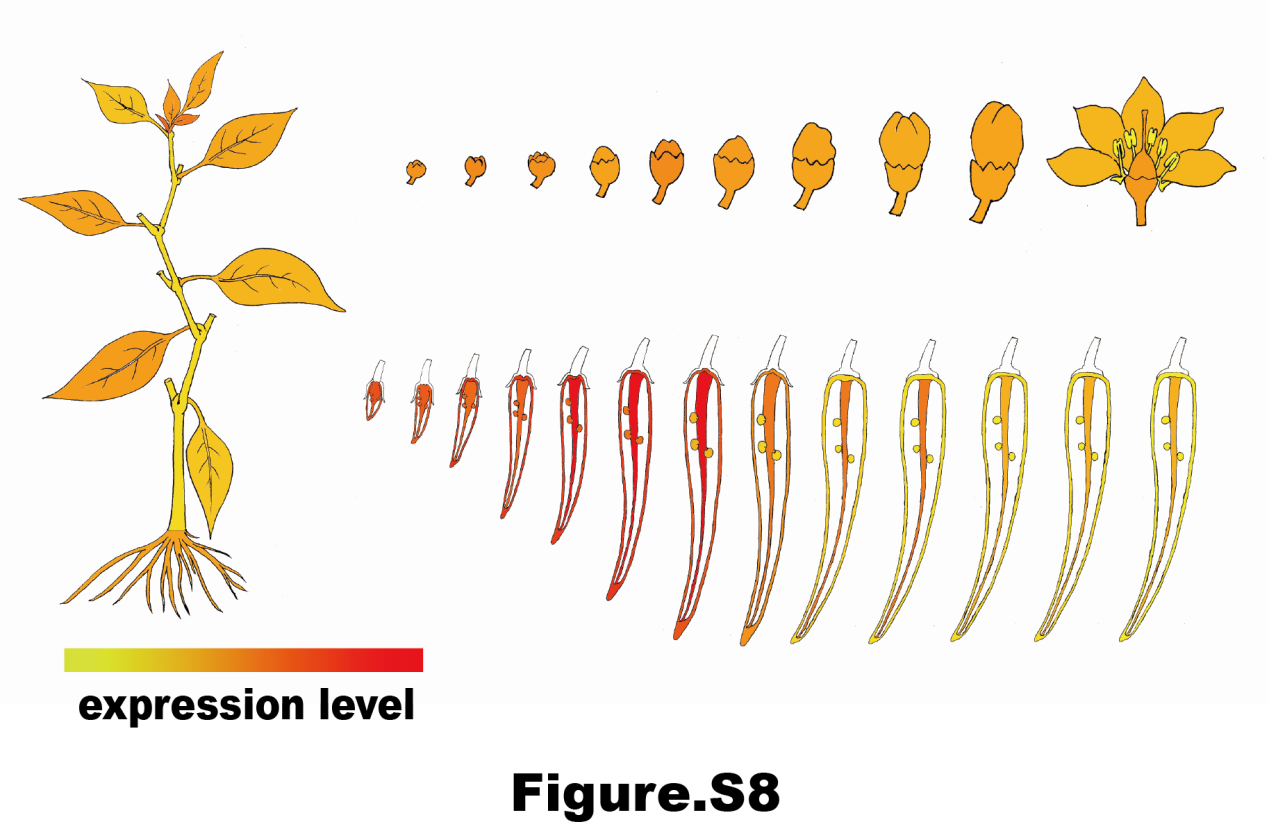
**

**Figure S14. Expression level of *Canq10g001705* in different tissues (Liu et al., 2017).**


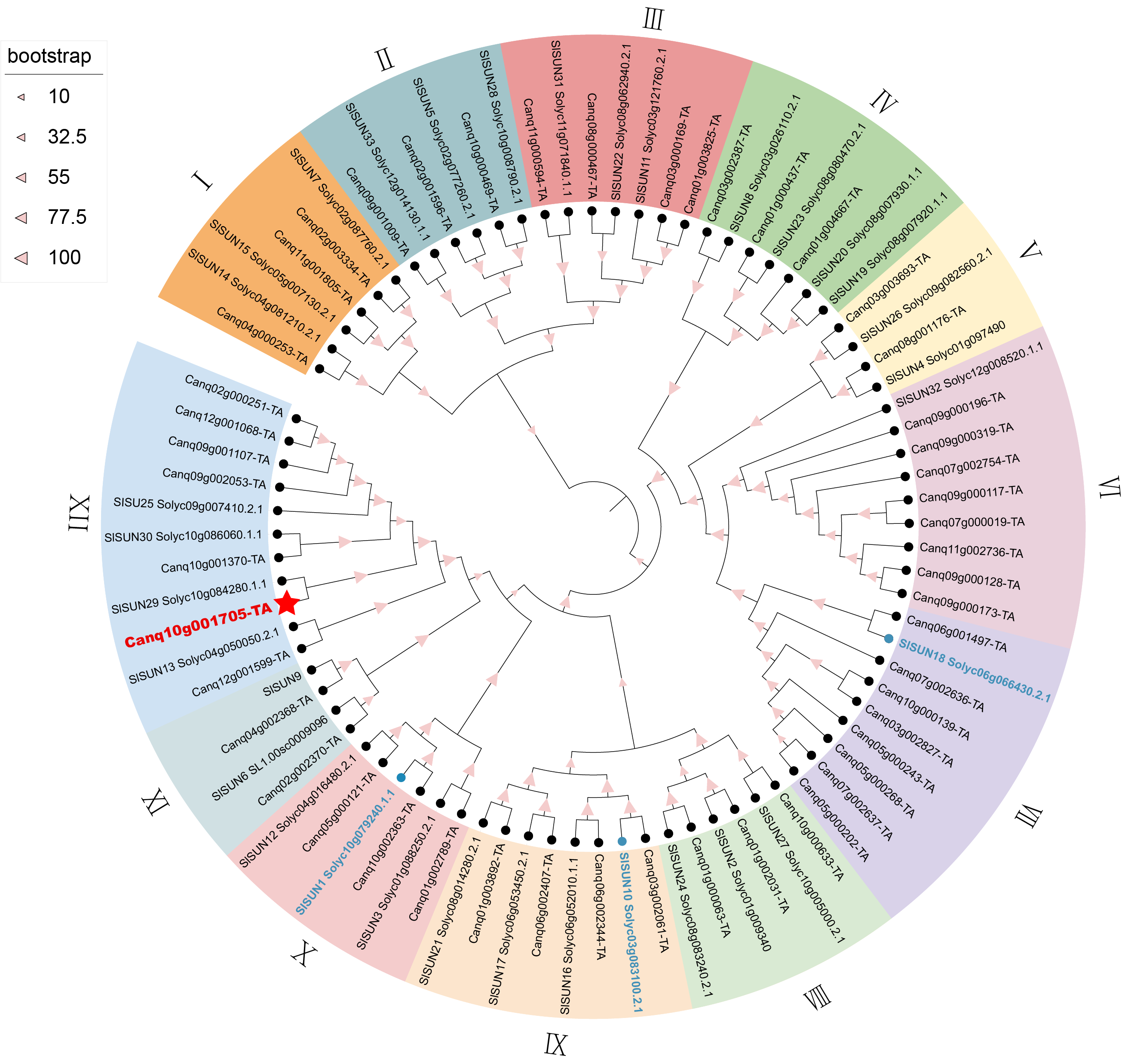


**Figure S15. Analysis of the phylogenetic tree of *Canq10g001705* (*CaSUN29*) and other pepper IQD genes and tomato *SUN* genes.**

The blue-marked *SUN* family genes of tomato have been identified as being associated with fruit shape regulation.

**
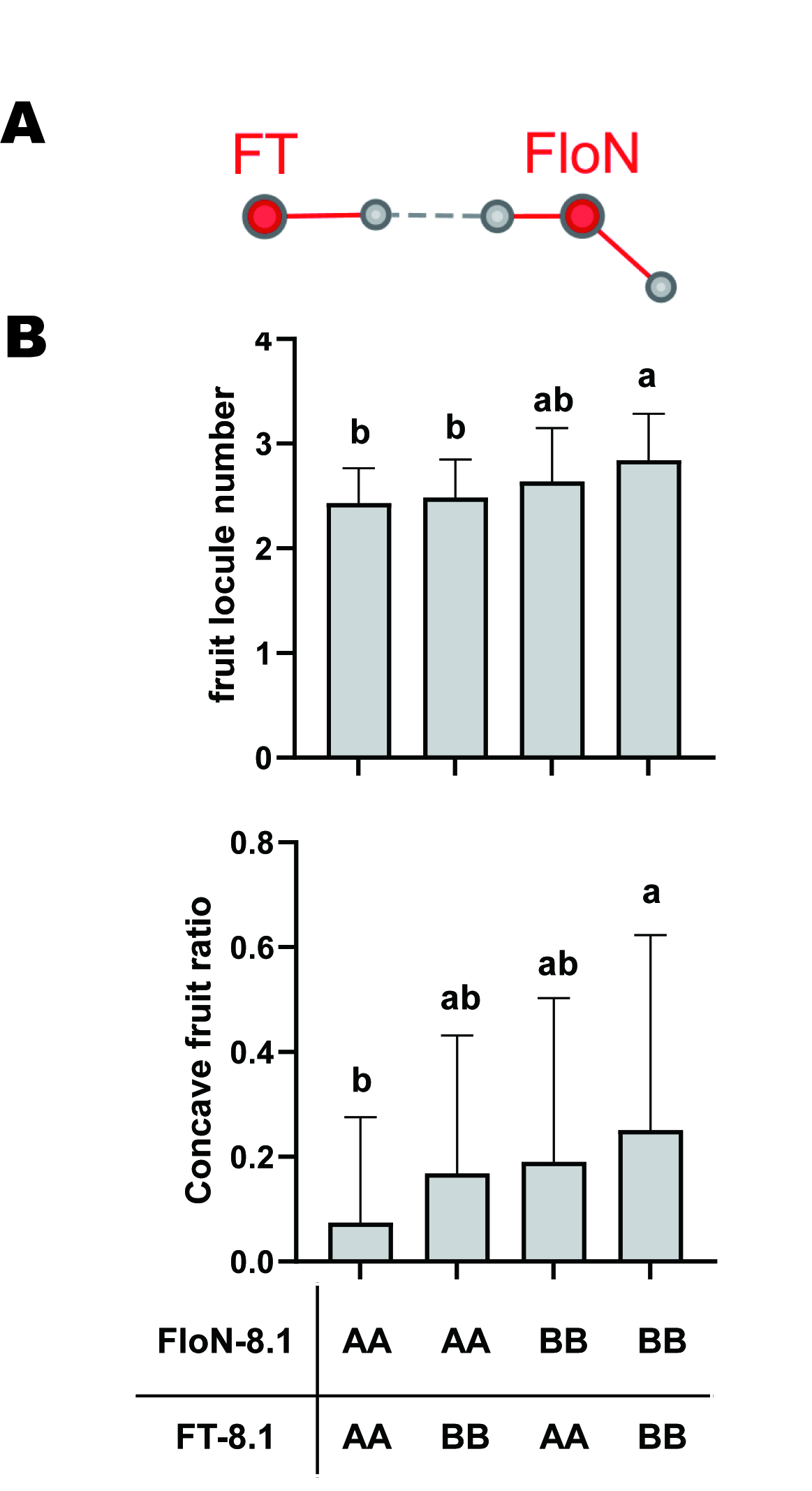
**

**Figure S16. Phenotypic variance of linked loci for fruit tip and fruit locule number.**

(A)Subnetwork of the fruit tip and fruit locule number. (B) Combinatorial effects of *FT-8.1* and *FloN-8.1* to concave fruit ratio and fruit locule number, respectively. AA: BVRC1 genotype. BB: BVRC25 genotype.


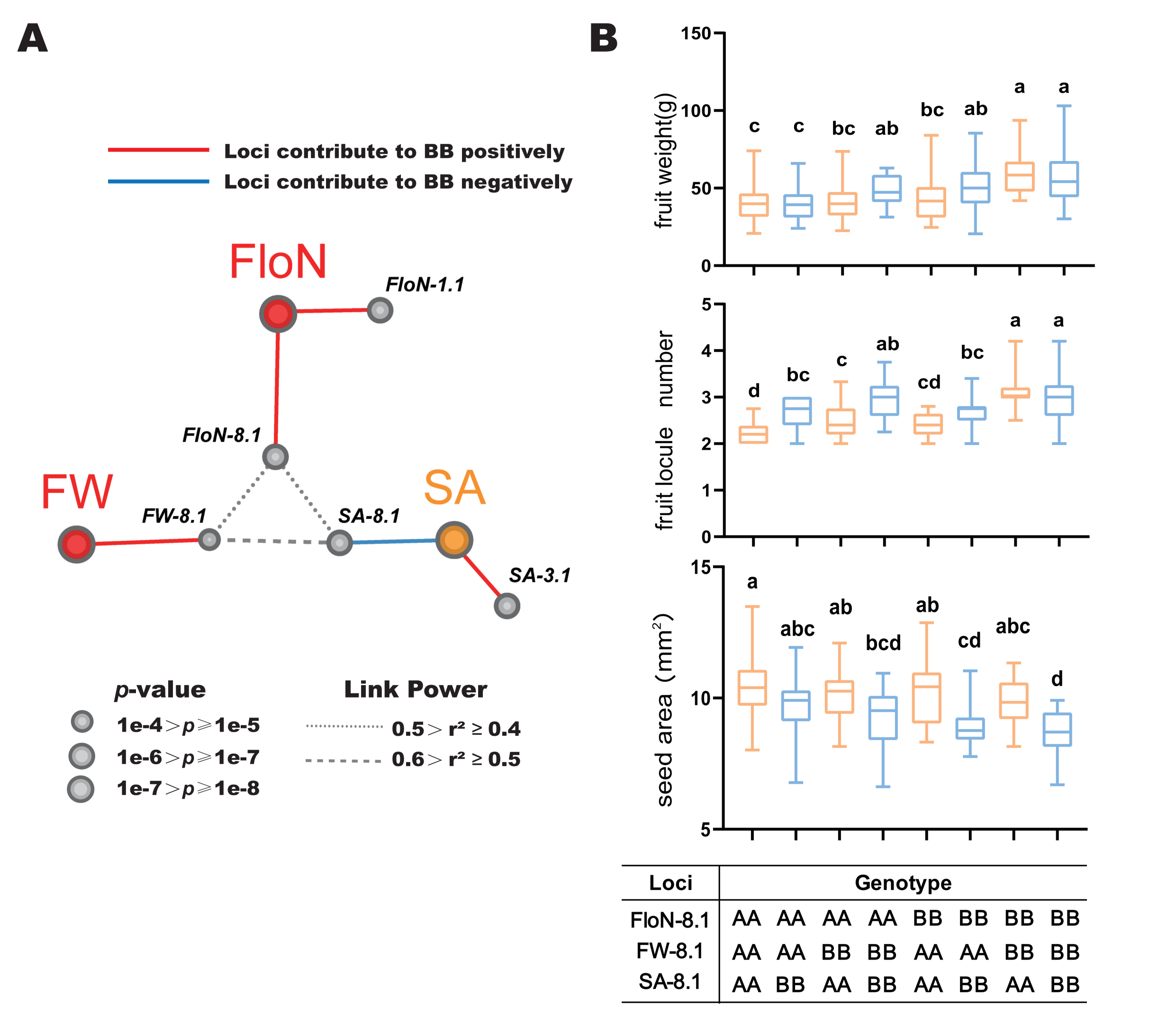


**Figure S17. Linkage and recombination among the loci based on the fruit weight, fruit locule number, and seed area.**

1. Subnetwork of fruit weight, fruit locule number, and seed area. (B) Effects of different genotype combinations at loci *FW-8.1*, *FloN-8.1*, and *SA-8.1* on fruit weight, fruit locule number, and seed area. Tukey’s honestly significant difference (HSD) test was used to identify significant differences among multiple groups, and different letters above the boxes indicate statistically significant differences, *P* < 0.05.

**SI References**

Liu, F., Yu, H., Deng, Y., et al. (2017). PepperHub, an Informatics Hub for the Chili Pepper Research Community. Mol Plant 10:1129-1132. 10.1016/j.molp.2017.03.005.
